# DNA Modifications: Function and Applications in Normal and Disease States

**DOI:** 10.3390/biology3040670

**Published:** 2014-10-22

**Authors:** Vichithra R. B. Liyanage, Jessica S. Jarmasz, Nanditha Murugeshan, Marc R. Del Bigio, Mojgan Rastegar, James R. Davie

**Affiliations:** 1 Department of Biochemistry and Medical Genetics, Manitoba Institute of Cell Biology, University of Manitoba, Winnipeg, MB R3E 0J9, Canada; E-Mails: umbatuwi@myumanitoba.ca (V.R.B.L.); murugesn@myumanitoba.ca (N.M.); Mojgan.Rastegar@med.umanitoba.ca (M.R.); 2 Department of Human Anatomy and Cell Science, University of Manitoba, Winnipeg, MB R3E 0J9, Canada; E-Mails: JJarmasz@mich.ca (J.S.J.); Marc.Delbigio@med.umanitoba.ca (M.R.D.B.); 3 Department of Pathology, University of Manitoba, Winnipeg, MB R3E 3P5, Canada

**Keywords:** DNA methylation, 5-methylcytosine, 5-hydroxymethylcytosine, methyl binding proteins, teratogens, brain development, neurological disorders, biomarkers, diabetes

## Abstract

Epigenetics refers to a variety of processes that have heritable effects on gene expression programs without changes in DNA sequence. Key players in epigenetic control are chemical modifications to DNA, histone, and non-histone chromosomal proteins, which establish a complex regulatory network that controls genome function. Methylation of DNA at the fifth position of cytosine in CpG dinucleotides (5-methylcytosine, 5mC), which is carried out by DNA methyltransferases, is commonly associated with gene silencing. However, high resolution mapping of DNA methylation has revealed that 5mC is enriched in exonic nucleosomes and at intron-exon junctions, suggesting a role of DNA methylation in the relationship between elongation and RNA splicing. Recent studies have increased our knowledge of another modification of DNA, 5-hydroxymethylcytosine (5hmC), which is a product of the ten-eleven translocation (TET) proteins converting 5mC to 5hmC. In this review, we will highlight current studies on the role of 5mC and 5hmC in regulating gene expression (using some aspects of brain development as examples). Further the roles of these modifications in detection of pathological states (type 2 diabetes, Rett syndrome, fetal alcohol spectrum disorders and teratogen exposure) will be discussed.

## 1. Introduction

Some argue DNA’s discovery to have been 145 years ago, with the more recognized discovery being 70 years ago, thanks to the works of Avery, MacLeod, McCarty, Watson, and Crick [1]. Since then, over a million published works can be found on the subject of DNA, which is the instructions manual of life [2]. DNA encodes our genes (also known as the genotype) and has been completely sequenced in the “Human Genome Project” as of 2003 [3]. Genes however are not always expressed, DNA is not always transcribed into ribonucleic acid (RNA), and RNA is not always translated into proteins. This complex network of gene regulation can partly be explained by epigenetics. The term was first coined in 1942 by Conrad Waddington, defining it as “*causal interactions between genes and their products which bring the phenotype into being*”. Epigenetics represents the many inheritable chemical “marks” found on and surrounding the genome that influence gene expression in a controlled and selective manner. DNA methylation and histone post-translational modifications (PTMs) are two epigenetic mechanisms involved in the regulation of gene expression [4]. DNA methylation was initially found and extensively studied in prokaryotic DNA since the 1960s. In 1977, 5-methylcytosine (5mC) was identified in eukaryotic DNA by Razin and Cedar [5,6,7]. Since their discovery, close to 33,000 published works can be found on DNA methylation, forever changing genetic and chromatin based research [8].

Epigenetic changes play a major role in biological processes at the level of chromatin structure and organization. DNA is packaged in the nucleus as a 10 nm diameter nucleosomal fiber (chromatin) and in higher-order chromatin structures, with the basic repeating unit being the nucleosome in which DNA is wrapped around a core of histone proteins. Access to DNA by various cellular machineries is granted by a series of epigenetic processes that change the high-order structure of chromatin. These epigenetic processes often entail dynamic chemical modifications to DNA or to the histones. Chemical modifications of histones include enzymatic methylation, acetylation, phosphorylation, ubiquitination, and sumoylation [9]. The addition of an acetyl group on a histone (predominantly on the N-terminal tail) is performed by lysine acetyltransferases (KATs), whereas its removal is performed by histone deacetylases (HDACs). Acetylation of histones generally allows for an “open” state of chromatin (termed euchromatin), exposing DNA and allowing transcription to occur. Methylation of histones may be either an activating or a silencing mark, depending on the specific amino acid affected. Interestingly there is “cross-talk” among the histone PTMs and DNA methylation/demethylation machinery, which is mediated by proteins that bind to the proteins involved in these modifications. For example, histone H3 trimethylated at lysine 4 (H3K4me3; an active mark) located at the 5' end of genes binds to recruit tumor suppressor inhibitor of growth protein 1b, which interacts with growth arrest and DNA damage protein 45a (GADD45) [10]. GADD45 mediates gene-specific DNA demethylation by recruiting DNA repair enzymes. The trimethylated H3K4 mark also recruits KATs, which result in nucleosomes with this mark having an increased steady state level of histone acetylation and reduced level of 5mC [11,12].

Collectively, DNA methylation is a critical player in many biological aspects, which are not limited to gene expression regulation. The knowledge on DNA methylation is rapidly advancing, and thorough understanding of its diverse functions is required to decipher the mechanisms by which alterations in DNA methylation contribute to human development and diseases. In this review we summarize several important topics in relation to the role of DNA methylation in biology and human diseases. In the second section of the review we will discuss how DNA methyl modifications are generated and relative locations of these modifications within the genome. In the third section, the role of DNA methylation and the role of two important DNA methylation-associated epigenetic modulators MeCP2 and CCCTC-binding factor (CTCF) in different biological processes will be discussed. In the fourth section, we will talk about the different types of methyl binding proteins (MBPs) and their role in DNA methylation-related biological processes and how alterations in DNA methylation and MBPs contribute to human diseases. This section will be followed by a detailed discussion on the effects of teratogens on DNA methylation in the brain, as well as the association of DNA methylation and neurodevelopmental disorders. In the finalsection, we will briefly evaluate the potential of DNA methylation as a biomarker for human diseases.

## 2. DNA Methylation

### 2.1. Five-Methylcytosine: “The Fifth Base”

**Figure 1:**
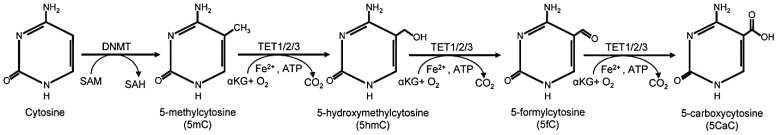
Generation of DNA methyl modifications. Cytosines are converted to 5-methylcytosine (5mC) by DNA methyltransferase (DNMT) enzymes through transfer of a methyl group from *S*-adenosyl methionine (SAM). Ten Eleven Translocation (TET) enzymes catalyze the oxidation of 5mC to 5-Hydroxymethylcytosine (5hmC) through a chemical reaction which involves *alpha*-ketoglutarate (αKG), Oxygen (O_2_), Adenosine triphosphate (ATP) and Fe^2+^. Similar reactions further oxidize 5hmC into 5-formylcytosine (5fC) and 5-carboxycytosine (5CaC).

DNA methylation is the focus of this review and represents “*the inheritable changes in gene expression that do not involve DNA sequence changes*” [4]. DNA methylation plays biological roles in genomic imprinting, reprogramming and stability, cellular differentiation, X-chromosome inactivation (XCI), transposon silencing, RNA splicing, and DNA repair [13,14,15]. The family of enzymes responsible for the covalent addition of the methyl group on the fifth carbon of cytosine (considered the “Fifth Base”) are DNA methyltransferases (DNMTs), with the methyl group donor generally being *S*-adenosyl methionine (SAM) (Figure 1). DNMT1, which is found in undifferentiated and differentiated cells, contributes to the maintenance of DNA methylation during cellular replication when both strands of DNA are separated and copied. DNMT1 preferentially binds to hemi-methylated DNA and copies the DNA methylation of the parental strand to newly replicated strand [16,17,18]. The *de novo* DNMTs are DNMT3A, DNMT3B and co-factor DNMT3L (DNA methyltransferase-like protein), which methylate DNA during embryogenesis and in differentiated cells, and are highly expressed in embryonic stem cells (ESCs). DNMT3B is essential for early embryogenesis, while DNMT3A is needed late in embryogenesis and found in differentiated cells. DNMT3L lacks a catalytic domain and is proposed to play the role of co-factor, enabling the *de novo* methylation function of DNMT3A and DNMT3B [16]. Because epigenetic marks are inheritable, the classic DNA methylation model labels DNMT1 as the enzyme which transfers the established DNA methylation patterns from the parental strand onto the daughter strands, therefore allowing the inheritance to occur and be subsequently maintained [18]. When DNMT1 does not effect this process, due to its absence from the nucleus, it is termed “*passive demethylation*” [15,19]. However, this model has recently been contested [20]. DNMT3A/B are gradual acting and highly expressed in somatic cells while their co-factor DNMT3L is not. This suggests that DNMT3L drives the *de novo* function of DNMT3A/B during embryogenesis only, while in somatic cells, DNMT3L is not expressed, and so DNMT3B actually stimulates DNMT3A. Once stimulated, DNMT3A/B anchor themselves to nucleosomes with DNA methylation, which is said to promote protein stability. Hypomethylation of nucleosomes disables the anchoring of DNMT3A/B, with the free DNMT3A/B being rapidly degraded. This is said to protect the genome from random *de novo* DNA methylation. The selective binding of DNMT3A/B suggests a regulatory role in the maintenance of DNA methylation. DNMT1 will copy the methylation patterns onto rapidly replicated hemi-methylated DNA. If incomplete, nucleosome anchored DNMT3A/B will “proof-read” and replete the pattern, thus propagating the inheritable methylated state [20,21,22].

DNMT1 is aided in its function by multiple proteins. Ubiquitin-like PHD and RING finger domains 1 (UHRF1) associate with DNMT1 at the replication fork. More specifically, proliferation cell nuclear antigen (PCNA) localizes DNMT1, and UHRF1 aids by binding hemi-methylated DNA and is then displaced in order for DNMT1 to specifically bind. This is accomplished by UHRF1’s many binding domains: an ubiquitin-like domain, an SRA domain (SET and RING domain), a RING domain with E3 ligase activity, a Tandem Tudor domain, a PHD domain, and an ADD-like domain. The SRA domain preferentially recognizes hemi-methylated DNA and binds securely via its base flipping mechanism, with the target nucleotide being 5mC. Tandem Tudor and PHD collectively bind histone PTMs (H3K9me3, H3K9me2, and H3K4me0) [9,20,23]. This suggests that DNA methylation and histone methylation collectively work together to ensure proper *de novo* DNA methylation and that UHRF1 faithfully ensures the transmission of DNA methylation during mitosis [9]. This is further supported by the fact that DNMTs are known to interact with histone methyltransferases, such as SUV39h1, Setdb1 and G9a [23]. However, UHRF1s mechanism has not been fully investigated, and others postulate that UHRF1’s RING domain ubiquitinates H3K23, which serves as a mark for DNMT1 to be recruited [23,24]. In addition, DNMT3L’s role as a co-factor also suggests the existence of a relationship between DNA methylation and histones. DNMT3A, DNMT3A, and co-factor DNMT3L all contain an ADD (ATRX-DNMT3-DNMT3L) domain, which is known to bind methylated or unmodified histones via its PHD domain within ADD. More specifically, DNMT3A and DNMT3L are capable of recognizing and binding unmethylated H3K4 and trimethylated H3K9. If H3K4 is trimethylated (H3K4me3), the PHD domain of ADD will not be able to bind [9]. This too fuels the coupling of DNA methylation and histone methylation, which has been recently summarized in *Rose and Klose’s 2014* review [23].

Therefore, when considering DNA methylation, it is critical to know that a variety of accessory proteins and modifying enzymes are operational in establishing and maintaining DNA methylation. Histone PTMs also play a role by blocking or promoting DNA methylation via specific histone acetylation or methylation marks, and are themselves regulated by KATs and HDACs.

DNA methylation occurs at approximately 70%–80% of CpG dinucleotides. Concentrated regions of CpG dinucleotides (CpG islands), which tend to be located within gene promoters, near transcription start sites, and in enhancer regions, are generally not methylated [13,17,23]. The exception is during development and within certain tissues, where a number of CpG islands are methylated, thereby turning genes off [13,16]. An example of this is CpG islands in somatic cells. They have been found to be methylated at a paternal (allele-specific imprinted genes) and maternal (X chromosome inactivation) level [16]. However, in mouse ESCs, approximately 800 CpG islands were found which contain 5hmC. Examples of genes with 5hmC methylated CpG islands include *Zfp64* and *Ecat1* [25]. Enzymatic proteins containing CXXC (Cys-X-X-Cys chromatin-associated binding domain) domains (e.g., DNMT1) bind unmethylated CpGs, while those with a methyl-CpG-binding domain (MBD; e.g., methyl-binding proteins, which are discussed in Section 3.1.2), bind mainly to methylated CpGs [9]. Non-CpG methylation, which is when guanine (G) is replaced with adenine (A), thymine (T) or cytosine (C), has also been identified in undifferentiated human embryonic stem cells (hESCs). 5mCpG remains to be the most prevalent in both un- and differentiated cell lineages; however, 5mCpA appears to be more prevalent in undifferentiated hESCs, decreasing as differentiation occurs. It is thereby thought to play a major role in cellular development [13]. However, 5mCpA has also been found in oocytes and in adult brain [16]. It has also been associated with *de novo* DNMT3 as the enzyme responsible for its methyl group addition, making the dynamics of DNA methylation even more complex [14].

### 2.2. Five-Hydroxymethylcytosine: “the Sixth Base”

Apart from 5mC, other DNA methylation marks exist and are rapidly becoming targets in “*active demethylation*” research in order to uncover their biological function [15,16,26]. “*Active demethylation*” represents the enzymatic removal or modification of the methyl group of 5mC [19]. The methyl group on the fifth carbon of the cytosine residue within the CpG can be oxidized by the ten-eleven translocation (TET) dioxygenase family, utilizing molecular oxygen as a substrate and therefore creating the “sixth base”: 5-hydroxymethylcytosine (5hmC) (Figure 1). Abundant levels of 5hmC can be found in the central nervous system (CNS) and in embryos of both humans and rodents, and is generally found near transcription start sites, making it essential for proper development [15,16]. Moreover, higher 5hmC enrichment was reported within gene bodies (intragenic regions) of actively transcribed genes in mouse ESCs [27] and enhancers in human ESCs [28]. The TET family of enzymes is comprised of TET1, TET2, and TET3, where TET1 and TET3 are known to bind CpG sequences via their CXXC domain [16,19]. The TET family have also been found to further oxidize 5hmC into 5-carboxylcytosine (5caC) and 5-formylcytosine (5fC) with the help of ATP (Figure 1) [16,29]. Deamination of 5hmC by the activation-induced cytidine deaminase/apolipoprotein B mRNA-editing, enzyme-catalytic, polypeptide (AID/APOBEC) family of deaminases can also occur, forming 5-hydroxymethyluracil (5hmU). This led to the hypothesis that 5hmC is an active demethylation mark, acting as an “initiation step” for base excision repair (BER) systems, which return 5mC to a regular unmethylated cytosine residue and therefore promote activation. Once 5fC and 5caC, are formed, they destabilize the *N*-glycosidic bond and subsequently encourage thymine DNA glycosylase (TDG) and methylated DNA binding domain-containing protein 4 (MBD4) glycosylases to initiate BER by removing the modified base and forming an apurine/apyramidine site (AP site). In the case of 5hmU, AID/APOBEC family of deaminases trigger BER and form an AP site as well. AP sites are toxic and need to be replaced with a base. AP endonuclease 1 (APEX1) will cleave the AP site and allow DNA polymerase to re-insert the appropriate base, in this case cytosine. In ESCs, 5hmC levels dominate that of 5fC and 5caC levels, implying that TET expression is strategically controlled [15,19,30,31,32]. However, researchers are still investigating the BER-mediated active demethylation mechanism with the hopes of finding increasing evidence to support it.

TET activity has also been tied to ascorbic acid (vitamin C), which acts as a cofactor in at least eight different enzyme reactions (including two related to carnitine synthesis) and is an antioxidant. Vitamin C has been linked to DNA demethylation in mouse ESCs. The catalytic site of TET requires Fe^2+^ and 2-oxoglutarate to oxidize 5mC. Vitamin C is postulated to be an additional co-factor for TET, possibly playing a role as a regulator and enhancer of TET activity. In the presence of vitamin C, TET primarily increases global oxidization of 5mC and promotes active demethylation [32,33,34].

## 3. Role of DNA Methylation in Biological Processes

As a major epigenetic mechanism, DNA methylation is involved in a vast array of biological processes. Examples of these processes include gene expression (transcriptional activation and repression), RNA splicing, genome organization, imprinting, and X chromosome inactivation (XCI), which are described in detail below.

### 3.1. Gene Expression

Before the discovery of secondary modifications of 5mC such as 5hmC, 5fC, and 5caC, the conventional role of DNA methylation in gene expression was believed to be repressive when 5mC is present at promoter regions [35]. The association of 5mC with alternative splicing also challenges the general repressive role of 5mC. Moreover, the association of 5hmC methyl marks with active chromatin regions [27,36] has challenged the general concept. DNA methylation can mediate transcriptional repression through three broad mechanisms: (1) direct hindrance of transcriptional activation; (2) recruitment of repressive protein complexes; and (3) cross-talk with histone PTMs. These mechanisms gain further complexity by the involvement of increased nucleosome compaction, prevention of the binding of transcription factors, recruitment of methyl binding proteins (MBPs), and prevention of the binding of chromatin remodeling complexes. 

#### 3.1.1. Direct Hindrance of Transcriptional Activation

DNA methylation (5mC) at promoter regions of genes may result in transcriptional repression through the prevention of the binding of transcription factors due to steric hindrance and increased nucleosome compaction. However, 5hmC favors transcriptional activation and thus questions the concept of the hindrance of transcriptional activation through DNA methylation. Many transcription factor binding sites harbor CpGs within them (e.g., CREB/ATF, E2F, c-MYC and NF-κB) and methylation at these CpGs hinders their binding [37]. Moreover, methylation of the CpGs adjacent to the transcription factor binding sites can also prevent their binding. For example, while the methylation of the CpGs within the Sp1/Sp3 transcription factor binding site does not affect its binding, methylation of the CpGs adjacent to the Sp1/Sp3 binding site prevents binding [38].

A correlation between DNA methylation and nucleosome dynamics has been demonstrated in the literature. DNA methylation influences many aspects of nucleosome dynamics including nucleosome positioning, stability [39] and structure [40]. The nucleosomal DNA is methylated in a 10 bp periodicity. Methylation at nucleosomal DNA is much higher than the flanking DNA, therefore, nucleosome positioning influences the methylation at the flanking DNA sequences [41]. In the case of the nucleosome structure, increased methylation leads to topological changes and more compact/tighter wrapping of the nucleosomal DNA around the nucleosome [40]. DNA methylation mediates more compact and rigid nucleosome positioning and thereby leads to a closed chromatin structure [42].

For these reasons, treatment with DNA demethylating agents or DNA methylation inhibitors such as 5-aza-2ꞌ-deoxycytidine (Decitabine), 5-azacytidine, and Zebularine results in a more open chromatin structure leading to transcriptional activation. Interestingly, depletion of DNA methylation by DNA demethylating agents has also been shown to result in increased localization of histone variants such as H2A.Z which causes nucleosome disassembly and supports transcriptional activation [43].

#### 3.1.2. Recruitment of Protein Complexes

The recruitment of DNA binding protein complexes including MBPs, co-repressor complexes and chromatin remodeling factors is a well-known mechanism for gene repression, mediated through DNA methylation. Once recruited to chromatin, these protein complexes are capable of mediating gene repression independently or in concert with cross-talks between histone PTMs. The recruitment of MBPs, such as MeCP2 and other proteins which contain a MBD, is a well-established mechanism of DNA methylation-mediated transcriptional repression or silencing [35,44]. The MBPs can recruit co-repressor complexes, which contain repressor proteins such as HDACs and SIN3. However, the role of MBPs in methylation-mediated transcriptional regulation is rather paradoxical because of the dual nature of the binding of these proteins to methylated DNA. For instance, 5hmC methylation was previously shown to prevent the binding of MeCP2 and was implicated in promoting the role of MeCP2 in transcriptional repression [45]. However, recent studies show that MeCP2 also binds to 5hmC at transcriptionally active chromatin domains [36] and that MeCP2 is an interacting protein partner of TET1 protein [46], suggesting its role in transcriptional activation. The precise role of MeCP2 binding to 5hmC was challenged by a recent study showing the repression of the *GAD1* and *RELN* genes in association with the binding of MeCP2 to 5hmC at their promoters in the cerebellum of autistic patients [47].

#### 3.1.3. Cross-Talks with Histone PTMs

The cross-talks between DNA methylation and histone PTMs can occur in two ways [35]. First, the DNA methylation established by DNMTs and/or TET proteins can lead to the recruitment of MBPs and other transcription regulatory proteins. These proteins can recruit the “writers” of histone PTMs followed by the recruitment of “readers” and/or “erasers”. Secondly, histone PTMs can directly or indirectly recruit the methyl writers (such as DNMTs) to establish DNA methylation [35].

Despite the significant advances made with regard to deciphering the role of DNA methylation in regulating gene expression, the presence of multiple methyl modifications, methyl binding proteins and interacting partners have complicated the perspective. Hence, the precise role of each methyl modification is still controversial and requires further analysis.

### 3.2. RNA Splicing

The role of promoter DNA methylation in gene expression regulation is well established. Recent knowledge concerning intragenic (or gene body methylation) and its role in alternative splicing have caused a paradigm shift in the conventional role of DNA methylation in transcription. Many recent studies have demonstrated the enrichment of DNA methyl marks within exons in contrast to the nearby intronic regions [48,49,50]. Moreover, there are subtle differences in the CpG density as well as methylation density between splice donor and acceptor sites. In this case, the donor sites have higher CpG density but are hypomethylated while the acceptor sites have less abundant CpGs and are hypermethylated [50]. The involvement of DNA methylation in splicing was further explained by its role in exon-intron recognition. Higher DNA methylation levels are observed in alternate exons [51] and spliced exons [49].

A handful of regulatory proteins, of which the chromatin binding is methylation-dependent, have been linked to the methylation-dependent alternative splicing.

#### 3.2.1. Role of MeCP2

In human fetal lung fibroblast cells (IMR-90), MeCP2 binding to methylated DNA within alternate exons contributes to the regulation of alternative splicing. DNA methylation at alternate exons drive the binding of MeCP2, while disrupted DNA methylation patterns caused ablation of the MeCP2 recruitment to these exons and subsequently results in elevated histone acetylation and alterations in exon skipping events [51]. MeCP2 recruits HDAC complexes, which reduces the steady state of the histone acetylation, resulting in an elongation rate that favors alternate exon inclusion. When HDAC activity is inhibited, MeCP2 levels are knocked down, or DNA methylation is inhibited, alternate exon exclusion will occur. The authors also demonstrated that MeCP2 is involved in the recognition of exons in splicing mechanisms [51]. The interaction of MeCP2 with spliceosome complex protein PRPF3 [52] and RNA binding protein YB-1 [53] as well as the altered alternative splicing events occurring in a MeCP2-mutant (Rett Syndrome) mouse model [53], further support the role of MeCP2 in splicing.

#### 3.2.2. Role of CTCF

Using CD44 gene splicing as a model for alternative splicing, CCCTC-binding factor (CTCF; a known insulator protein or a boundary element) was shown to be important in methylation-dependent co-transcriptional alternative splicing [54,55]. According to this model, hypomethylation of the CTCF binding site within exon 5 of the CD44 gene recruits CTCF onto exon 5, pauses RNA polymerase II (RNAPII), and subsequently causes inclusion of exon 5. In contrast, hypermethylation of the CTCF binding site prevents CTCF binding, which ultimately causes fast movement of RNAPII and exclusion of the weak exon 5. Coupling of RNAPII pausing to splicing through CTCF has also been demonstrated in the *Myb* gene [56]. CTCF is capable of mediating long-range interactions between intronic elements, promoter and upstream promoter enhancer elements, which leads to RNAPII pausing. Moreover, high RNAPII pausing index was found to be highly correlated with the recruitment of CTCF to upstream promoter elements and 5ꞌ untranslated regions (UTRs) [57].

#### 3.2.3. Genome Organization

DNA methylation and histone PTMs function together to maintain the structure of chromatin, which is ultimately represented in gene expression patterns within a cell. Through dynamics of DNA methylation and histone PTMs, either open or closed chromatin structures are obtained to aid transcriptional activation or repression, respectively. Therefore, DNA methylation plays a critical role in maintaining genomic organization. The role of DNA methylation in genomic organization can be further subdivided into different aspects such as modulation of chromatin architecture, maintenance of genomic stability and reduction of transcriptional noise (variability in gene expression) caused by spurious transcription. Hence, the context of DNA methylation as well as the context-dependent recruitment of methylation-related proteins, such as MBPs and CTCF, are important in maintaining the genomic organization.

#### 3.2.4. Modulation of Higher Order Chromatin Structure

The role of DNA methylation in regulating chromatin structure via euchromatin or heterochromatin is well established and extensively studied. Different methyl modifications contribute to chromatin structure in different ways. Euchromatin regions are generally enriched with 5hmC while heterochromatin domains are devoid of 5hmC in mouse ESCs [58]. Similarly, 5caC is accumulated in euchromatin regions in ovarian follicular cells [59]. Moreover, both 5fC and 5caC are found in repetitive microsatellite loci in mouse ESCs [60]. Once again, these studies highlight how DNA methylation is context-dependent.

MBPs function as chromatin architectural proteins and therefore contribute to genome organization [35]. The chromatin architectural role of MeCP2 has been extensively studied. MeCP2 is highly abundant in neurons with levels similar to histone proteins [61], and competes with histone H1 to bind to linker DNA [62]. Absence of MeCP2 or mutations that cause disruption of the MeCP2-chromatin bridge alter formation of higher order chromatin structures [63]. MeCP2 contributes to the formation and maintenance of the higher order structures through the formation of loops and bridges [64,65].

#### 3.2.5. CTCF and Genome Organization

CTCF also contributes to genome organization as a chromatin architectural protein. It has been referred to as the master genome organizer because of its diverse functions (discussed in detail in Section 4.2.1) [66]. CTCF mediates long distance interaction between different chromatin loci through the formation of chromatin loops [67,68]. Chromatin immunoprecipitation (ChIP), chromosome conformation capture studies, and chromatin interaction analysis by paired-end-tag sequencing (ChIA-PET) have revealed interactomes (protein-protein interactions) of CTCF within the genome which represent both inter- and intra- chromosomal interactions mediated through CTCF binding to chromatin [69,70]. Therefore, CTCF is involved in the formation of a genomic web of interactions.

#### 3.2.6. Nuclear Architecture

Another function of DNA methylation and associated proteins is in nuclear organization, which is an indirect representation of the genomic organization. Alterations in the nuclear architecture were observed in the absence of DNA methylation [71]. Nuclear architecture can be visualized as chromocenters, chromosome territories, and the size and shape of nuclei. Alterations in DNA methylation itself or the MBPs cause changes in the aforementioned features. For example, loss of MeCP2 in brain or overexpression of MeCP2 in neurons cause changes in the number and size of chromocenters, as well as size of the nuclei and nucleoli [72]. Impaired DNA methylation patterns cause reorganization of chromocenter territories [73].

#### 3.2.7. Transcription Noise

Recent studies show that gene body methylation (exons and introns) affects the distribution of DNA methylation, histone PTMs and the RNAPII recruitment, and subsequently affects molecular processes such as transcription and RNA splicing. Gene body methylation also reduces the transcriptional “noise” caused by spurious transcription [74]. However, this concept, which was based on the notion that DNA methylation is generally repressive, has been challenged by new understanding of the distribution of 5hmC within gene bodies [27]. Recruitment of MeCP2 to methylated repetitive elements was also shown to reduce transcriptional noise [61] and thus MeCP2 is being considered as genome-wide epigenetic modulator rather than a specific transcriptional regulator [75]. MeCP2 is able to bind to both 5mC and 5hmC [36]. It is theoretically capable of regulating transcriptional noise regardless of the methyl modification. Moreover, DNA methylation contributes to genomic organization through the suppression of the proliferation of transposable elements [76,77,78].

### 3.3. Imprinting

DNA methylation is an essential process in primordial germ cells (PGC). It establishes genomic imprinting and occurs during PGC reprogramming [79]. Several lines of recent evidence show that, during this process, 5mC is converted to 5hmC (which is functionally the same as demethylation) [80,81]. These studies highlight the roles of both 5mC and 5hmC methylation in genomic imprinting. Genomic imprinting involves silencing of alleles, allowing for preferential gene expression exclusively determined by the parent-of-origin and not the DNA nucleotide sequence itself. It has been established that alleles are silenced by selectively methylating the imprint control region (ICR) within a gene. This blocks ICR’s binding site potential and downstream-enhancer mechanisms. Differentially methylated regions (DMRs), which are also inherited from either the paternal or maternal chromosome, are located near imprinted genes as well, which in turn affects gene expression. Each paternal and maternal allele contains different DMRs, allowing for regulatory proteins like CTCF to bind DNA and interact with both the ICR and DMRs, allowing the formation of a “chromatin loop” and therefore protecting the allele from methylation [4]. The majority of the imprinted genes are clustered in imprinted domains and these genes harbor DMRs, which are characterized by the parent-of-origin-specific DNA methylation profiles [82]. Well known imprinted domains carrying specific DMRs include *IGF2/H19*, *DLK1/MEG3* and *SGCE/PEG10*. These DNA methylation profiles within the imprinted genes serve a good biosensor for the early exposure to environmental factors and insults as a measurement of the epigenetic memory of early events [82,83,84]. It also seems that the changes in DNA methylome at the imprinted genes might render susceptibility to imprinting-related diseases [85,86].

Several DNA methylation-related epigenetic factors are involved in the regulation of imprinted genes [84]. For example, the paternal *H19* requires the epigenetic modifiers DNMT1, DNMT3A, and DNMT3A. MeCP2 is known to regulate *Dlx5* and *Ube3a* [87,88]. CTCF functions as a boundary element or insulator for the *IGF2/H19* locus and is able to bind to the unmethylated DMR of the maternally inherited *IGF2/H19* locus. This binding creates an insulator region for the *Igf2* promoter and prevents the interaction between *Igf2* promoter and its enhancers [89]. Moreover, CTCF interacts with cohesin and is bound to a subset of imprinted genes such as *Magel2/Peg12* to which CTCF binds in an allele-specific manner [90]. Whether the binding of the epigenetic factors such as CTCF and MeCP2 to DMRs is dependent of the type of methyl modification is still unclear.

Another key player in imprinting is the more recently discovered maternal effect gene ZFP57. This gene codes for a zinc-finger binding protein, which is part of the Krüppel-associated box (KRAB) repressor protein family. ZFP57 is thought to recruit DNMT1 to the ICRs, thereby explaining mechanistically how DNMTs choose which allele is to be methylated. ZFP57 interacts with co-factor KAP1/TRIM28, which contains various domains that have epigenetic regulatory components by functioning as a scaffold: heterochromatin protein 1 (HP1) binding motif, plant homeodomain (PHD), and bromodomain (BRM) [91,92] These domains are known to bind modified histones. These in turn recruit chromatin modifying enzymes including nucleosome remodeling deacetylase (NuRD) complex, HP1, histone-lysine N-methyltransferase (SETDB1), UHRF1 and DNMTs [23,91,92]. Taken together, this paints a rather complex picture, depicting direct involvement of histone PTMs and chromatin-remodelling machinery in mediating DNA methylation at imprinted alleles. Particularly, methylated histones (H3K9me3 and H4K20me3) are thought to be associated with ICRs. H3K9 is trimethylated by Suv39h1 or Suv39h2 and DNA methyl binding proteins like MeCP2 are known to associate with Suv39h1/2. MeCP2 brings in DNMT1, therefore inducing CpG methylation and establishing heterochromatin [23,92].

### 3.4. X-chromosome Inactivation

Apart from genomic imprinting, XCI is another method to drive monoallelic gene expression. The X chromosome is home to roughly 1,000 genes [93]. In human females, having both X chromosomes expressed would double the dose of those genes, not only creating an imbalance between sexes, but also potentially being toxic to the cell [94,95]. During the late-blastocyst stage, one of the two X chromosomes in every cell is randomly chosen to undergo eternal silencing, termed XCI [96]. This allows for both males (XY) and females (XX) to have equal X-linked gene dosages, termed dosage compensation [93,94]. DNA methylation is believed to be important in the inactivation process as well as maintenance of the inactivated X-chromosome [97]. A basic difference between the active X-chromosome (Xa) and the inactive X-chromosome (Xi) is the level of DNA methylation. Xi has higher levels of DNA methylation, which maintain a compact chromatin structure. Moreover, the genes found within Xi tend to have methylated the CpG islands in contrast to the unmethylated CpG islands in Xa [98].

The human X chromosome contains an X inactivation center (XIC), which harbors two genes essential for XCI, *XIST* and *TSIX*. Both genes encode noncoding RNAs (ncRNAs). *XIST* ncRNA is over 19 kb and remains in the nucleus. *TSIX* ncRNA is greater than 30 kb and is complement to *XIST*. Although the choice of which X chromosome to inactivate is random, the inactivation process itself is not. In mammals, the initiation of XCI starts with the downregulation of *trans*-*TSIX* and the upregulation of *cis*-*XIST* on the soon-to-be inactive X chromosome (Xi). The *TSIX* levels will remain stable on the active X chromosome (Xa) and inhibit the transcription of *XIST*. *XIST* ncRNA will then travel across the Xi, coating it, and recruits the polycomb repressive complex 2 (PRC2) which will bind *XIST*, and in turn, recruit PRC1 and DNMTs to subsequently methylate the CpG islands of gene promoters. This gives Xi an overall condensed (heterochromatic) status identifiable at the histologic level and known as the “Barr body”. However, not all genes on the X chromosome are silenced. It is estimated that 15%–20% of X-linked genes escape silencing (the pseudo-autosomal region, PAR included), and roughly 10% of genes on XCI are only partially silenced [93,99]. There is also evidence suggesting direct contact between enhancer of zeste homologue 2 (EZH2) of PRC2 and DNMT3A/B, however contradictory evidence exists as well [100,101].

The *Xist* promoter itself is regulated by promoter methylation, and the *Tsix* noncoding RNA was shown to activate the DNMTs to mediate the DNA methylation at the *Xist* loci leading to the repression of *Xist* gene [102,103]. Moreover, *Tsix* has been shown to function together with CTCF to determine which X-chromosome will be inactivated [104]. Even though imprinting and XCI are theoretically different processes, they are linked to each other. Among the types of XCIs occur during mouse embryonic development, there are two types: imprinted XCI and random XCI. During mouse embryogenesis, the paternal X-chromosome is inactivated through the expression of paternal *Xist* and binding of this *Xist* transcript to the X-chromosome. This paternally inactivated X-chromosome is also called imprinted XCI. However, unlike the random XCI, it seems that DNA methylation does not play a significant role in imprinted XCI because during this stage of embryogenesis (morula stage) the genome undergoes DNA demethylation [105].

## 4. Methyl Binding Proteins: “The Methyl Readers”

**Figure 2:**
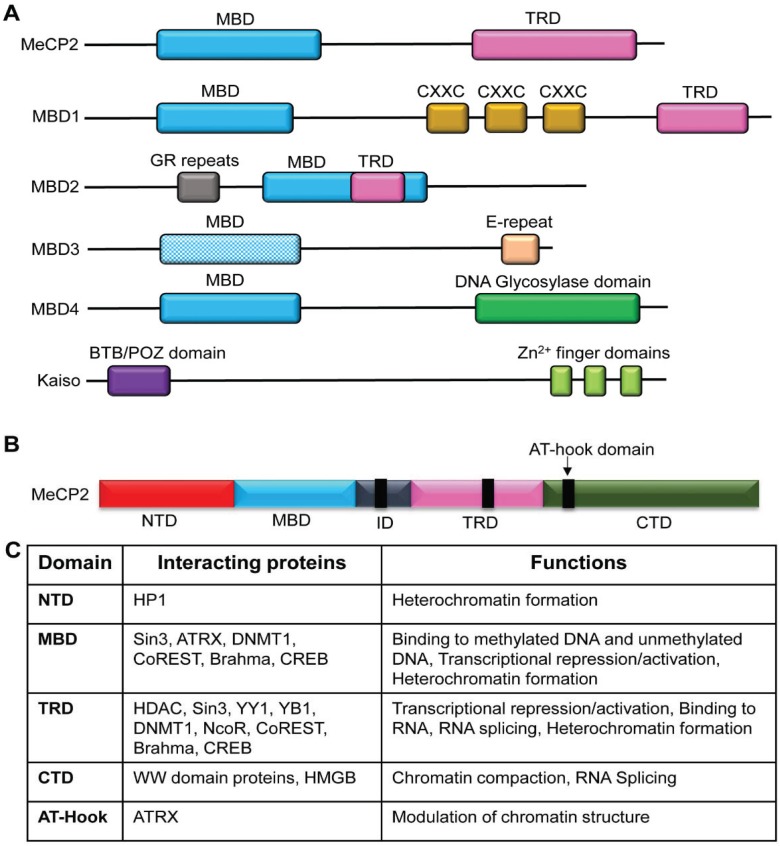
The structure of methyl-CpG-binding protein (MBP) family. (**A**) Comparison of structure of MBPs. The MBP family shares the characteristic methyl-CpG-binding domain (MBD), which enables the recognition and binding to methylated DNA. However, MBD3 is unable to bind to methylated DNA. The transcription repression domain (TRD) is found in MeCP2 and MBD1-2. Apart from MBD, the binding of specific MBDs to DNA can be mediated through CXXC zinc-finger (Zn^2+^) motifs, glycine and arginine residues (GR), and thymine glycosylase. The Kaiso family of proteins is characterized by a BTB/POZ domain and a triple zinc-finger domain. (**B**) Domains of MeCP2. Apart from the characteristic MBD, there are other domains, which are important in protein interactions and functions. Abbreviations: NTD: N-terminal domain; MBD: Methyl-CpG-binding domain; TRD: Transcription repression domain; ID: interdomain; CTD: C-terminal domain. (**C**) Interacting protein partners and functions of MeCP2. HP1: Heterochromatin Protein 1; ATRX: ATP-dependent helicase ATRX; DNMT1: DNA methyltransferase 1; HDAC: Histone deacetylase; CREB: cAMP response element-binding protein; HMGB: High-mobility group box.

As described previously, DNA methylation at specific loci is established by DNMTs and/or TET proteins. However, in order to interpret the methylation information established by these enzymes, a “methyl reader” is required, which can recognize, bind to, and recruit other protein complexes to methylated DNA. Methyl binding proteins or MBPs are characterized by their conserved methyl-CpG-binding domain (MBD) which basically allows them to recognize and bind to methylated DNA (see Figure 2A). The proteins that belong to MBP family include methyl-CpG-binding protein 2 (MeCP2), MBD1, MBD2, MBD3 and MBD4 [106]. However, despite the presence of the MBD domain, MBD3 is unable to bind to methylated DNA, presumably because of the presence of two extra amino acids (His-30 and Phe-34) within the domain [107]. However, MBD3 can still function as a transcriptional regulator similar to other MBPs through interactions with HDAC1 and MTA2 found in NuRD/Mi2 complex [107]. Apart from the proteins with MBD domain, Kaiso proteins, which lack a MBD domain, are also able to bind to methylated DNA through their Zinc (Zn) finger domains [108].

The overall structure of the MBPs is critical in their biological functions. For example, MeCP2 (considered to be the prototype protein of the MBP family) is comprised of two major domains: MBD and transcription repression domain (TRD). The MBD and TRD domains mediate binding to methylated DNA and transcriptional regulation, respectively. MeCP2 is a multifunctional epigenetic modulator and its major functions include transcriptional regulation (both transcriptional activation and repression), chromatin structure modulation and RNA splicing [109]. The diverse functions of MeCP2 are mediated by protein-protein and protein-DNA interactions, where these interactions are mediated through different domains of the MeCP2 protein (see Figure 2B,C).

The requirements for binding to methylated DNA differ from one MBP to another and are DNA sequence-dependent. Such sequence-dependent binding requirements should contribute to the unique binding to target gene promoters [110]. MeCP2 protein requires a single CpG with at least four adjacent A/T pairs [111]. MBD1 binds to a single CpG site in the context of TCpGG, CCpGA, GCpGG and CCpGC with varying degrees of efficiency [112]. The binding of MBD2 to methylated DNA is determined by the presence of either CpGG or CpGC sequences within the target gene promoter, with a higher affinity towards CpGG [113]. In contrast, the Kaiso proteins prefer binding to two symmetrical methylated CpGs in the context of CGCG [108]. Not only the underlying sequence, but also the type of methylation (5mC, 5hmC, 5fC or 5caC) determines the binding of MBPs to chromatin. Recent studies have determined the diverse patterns of MBP binding to methylated CpGs in different cell/tissue types and at different genomic loci (Table 1). Among the MBPs, MeCP2 has been shown to bind to both 5mC and 5hmC with high affinity and was shown to be a major 5hmC binding protein in the brain [36].

**Table 1:** Examples of proteins, which bind to methyl marks.

Methyl Mark	Methyl Binding Protein	Description: Cell/Tissue Type (Genomic Location)
5mC	MeCP2	Mouse ESCs, Mouse Neural precursors, Mouse Brain [114]
	MBD1	Mouse ESCs, Mouse Neural precursors, Mouse Brain [114]
	MBD2	Mouse Neural precursors, Mouse Brain [114]
	MBD3	E14 Mouse ESCs (*Fgf15*) [115]
	MBD4	E14 mouse ESCs (*Pax6*) [115]; Mouse ESCs, Neural precursors, Brain [114]
5hmC	MeCP2	Mouse Brain (*Active chromatin*); Mouse ESCs [114]
	MBD3	E14 mouse ESCs [116]
	MBD4	Mouse Neural precursors [114]
5fC	MBD3	E14 mouse ESCs (*Fgf15*) [115]
5mC	SIN3A	E14 mouse ESCs (*Pax6, Fgf15*) [115]
	CREB1	E14 mouse ESCs (*Pax6, Fgf15*) [115]
	SRSF2	E14 mouse ESCs (*Fgf15*) [115]
	SRSF3	E14 mouse ESCs (*Pax6*) [115]
5hmC	SRSF2	E14 mouse ESCs (*Fgf15*) [115]
	SRSF3	E14 mouse ESCs (*Pax6*) [115]
	CREB1	E14 mouse ESCs (*Pax6*) [115]
5fC	SIN3A	E14 mouse ESCs (*Fgf15*) [115]
	CREB1	E14 mouse ESCs (*Pax6, Fgf15*) [115]
	SRSF2	E14 mouse ESCs (*Fgf15*) [115]
	SRSF3	E14 mouse ESCs (*Pax6*) [115]
5CaC	CTCF	Mouse ESCs [114]
	EHMT1	Mouse ESCs [114]
	NCOR2	Mouse ESCs [114]
	PRP31	Mouse ESCs [114]
	DNMT1	Mouse ESCs [114]

Note: SIN3A, NCOR2 (corepressors), CREB1 (coactivators), SRSF2/3, PRP31 (splicing factors/mRNA processors); DNMT1 (DNA methyltransferase); EHMT1 (histone methyltransferase); CTCF (insulator/transcription factor/multifunctional protein).

Even though MBPs are characterized by their binding to methylated DNA, these proteins can also bind to unmethylated DNA. For instance, MeCP2 is able to bind to unmethylated DNA [117,118], which is mediated through MBD [62,119] as well as inter domain (ID), TRD and C-terminal domain (CTD) [119]. The CXXC3 zinc finger domain of MBD1 drives its binding to unmethylated CpGs [120]. A recent study also demonstrated the affinity of MBD3 towards both methylated and unmethylated CpGs, similar to MBD2 [121].

As discussed in Section 2, the biological functions of MBPs are diverse and include, but are not limited to, modulation of chromatin architecture, gene expression regulation and RNA splicing. Apart from that, MBPs also play a role in maintenance of DNA methylation. For example MBD4 is involved in maintenance of methylation and suppression of the mutations occurring at CpG sites [122]. Moreover, MeCP2 interacts with DNMT1 to perform DNA methylation maintenance [123].

### 4.1. DNA Methylation and MBPs in Human Diseases

Alterations of DNA methylation patterns are known to be associated with a variety of human diseases. DNA methylation is highly susceptible to environmental cues and environmental insults such as exposure to toxins, teratogens (see Section 5), diet (nutrient availability), and mental state (stress). It is a dynamic and heritable epigenetic modification, and thus alterations of methylation can be transmitted through generations, which are usually referred to as “*transgenerational epigenetic effects*” [124]. Moreover, single nucleotide polymorphisms (SNPs) are found in DMRs and many of these SNPs (CpG-SNPs) are associated with several human diseases such as type 2 diabetes [125]. CpG-SNPs contribute to the disease through either introducing a CpG or disrupt a CpG affecting the DNA methylation patterns, gene expression and changes in splicing. Furthermore, CpG-SNPs at the *Prodynorphin* gene were found to be associated with alcohol dependence [126]. DNA methylation at the promoter of specific genes or gene body methylation affecting splicing can contribute to human diseases. The involvement of DNA modifications in brain development and neurological disorders has been studied extensively and is described in Section 5.

**Figure 3:**
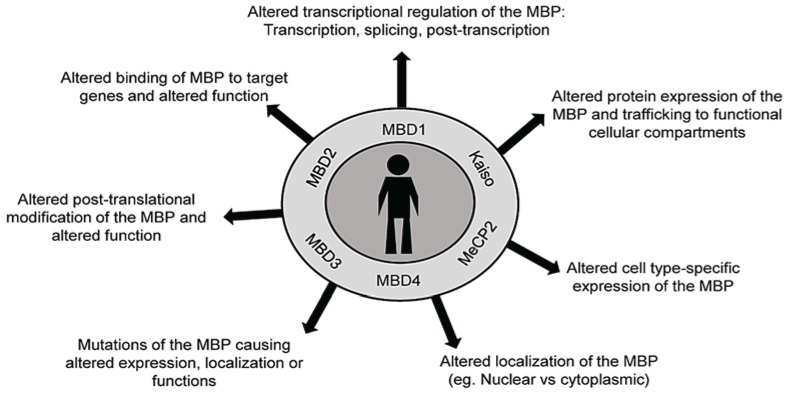
The ways alterations in methyl binding proteins (MBPs) cause human diseases. Major methyl binding proteins (MBPs) include Methyl binding domains 1, 2, 3, 4, (MBD1, MBD2, MBD3, MBD4), Methyl CpG binding protein 2 (MeCP2) and Kaiso proteins. The alterations in their expression, posttranslational modifications, functions, and localization can contribute to human diseases.

A major mechanism by which DNA methylation is involved in human diseases is through the involvement of MBPs. MBPs can cause human diseases through multiple ways (see Figure 3). They include any process which affects their expression and functions such as alterations in (1) transcriptional regulation (transcription, splicing, posttranscriptional regulation); (2) protein expression and trafficking to different functional cellular compartments; (3) expression in specific cell types; (4) localization (nuclear, cytoplasmic, nucleoli); (5) mutations causing impaired expression, localization or functions or binding to interacting proteins/DNA; (6) posttranslational modifications causing impaired function; and (7) binding to chromatin and target genes. The presence of such mechanisms in several human diseases including cancer, diabetes as a metabolic disorder, imprinting disorders, and immune system-related disorders are described in detail below.

#### 4.1.1. Cancer

Cancer was long regarded as a genetic disease caused by spontaneous mutations in the genome, but recent findings have showed that alterations in the epigenome are also important [127,128]. Correct methylation patterns are necessary for the appropriate binding of MBPs [129]. The precise roles of MBPs differ between the different types of cancers.

In the absence of MeCP2, a two-fold increase in the expression of genes that play a role in prostate cancer derived PC3 cell line survival, proliferation, migration, and invasion (e.g., *BAK1*, *Cant1*, *Cav1*, *CCND1*, *CD164*, *Enolase 2*, *RASSF1A* and *NTN4*) is seen [128]. In myeloma, regulation of *SPAN-XB* gene expression by binding of the MeCP2 protein to the promoter dictates cell proliferation [130].

*MBD2* mRNA levels may directly or indirectly play a role in the production and maintenance of DNA methylation of genes important in gastric carcinogenesis [131]. Three SNPs in the *MBD2* gene are associated with reduced risk of breast cancer [132]. Binding of the MBD2 protein with CCAAT/enhancer binding protein alpha (CEBPA) transcription factor enhances or suppresses expression of genes involved in liver cancer [133]. In uterine serous carcinoma (USC), deletion of a small segment of chromosome 19, which includes the *MBD3* gene, is a frequent occurrence [134]. In gastric carcinoma, decreased MBD3 mRNA levels are associated with dissociation of the NuRD complex and aberrant DNA methylation [131,135,136]. A polymorphism in* MBD4* Glu364Lys seems to influence protein stability as it is associated with increased susceptibility to cervical cancer [137].

Kaiso protein nuclear localization and binding to the promoter of E-cadherin leads to suppression of E-cadherin protein (involved in cell junctions), which in turn may be involved in breast cancer development [138,139]. Consociation of Kaiso proteins and p120-catenin in the cytoplasm of lung cancer cells is associated with its phenotypes. Dai *et al.*, further suggest that abnormal p120^ctn^ expression and cytoplasmic expression of Kaiso is also observed in lung cancer phenotypes. Kaiso proteins may have an oncogenic function in the cytoplasm of lung cancer cells and their localization is affected by p120^ctn^ which is its binding partner [140].

#### 4.1.2. Diabetes Mellitus

**Figure 4:**
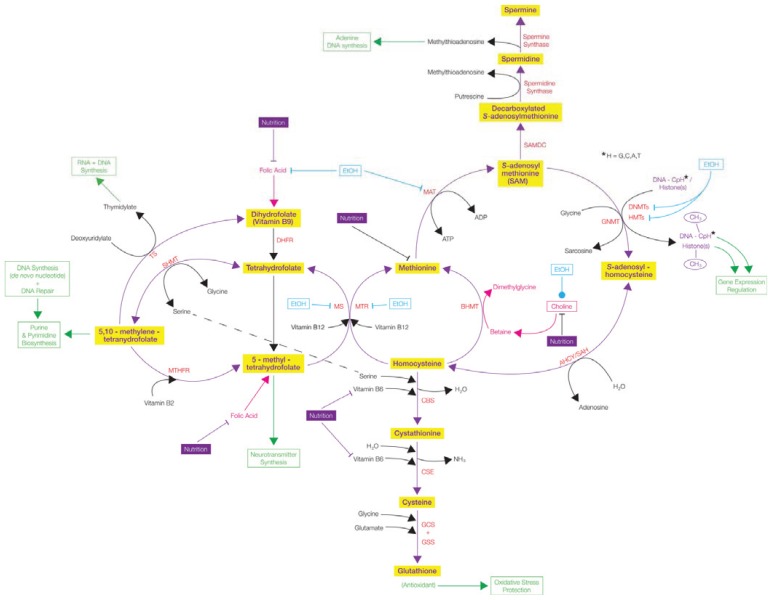
One carbon metabolism and the effects of alcohol (EtOH) and nutrition throughout the metabolical pathway. Ethanol is known to increase histone 3 (H3) acetylation, increase and decrease H3 methylation levels, and alter mitogen-activated protein (MAP) kinases. Ethanol can also have an effect on *S*-adenosylmethionine (SAM) biosynthesis, which is a methyl donor for DNA methyltransferases (DNMTs) and histone methyltransferases (HMTs), thereby having an effect on DNA methylation (generally hypomethylation). It also plays roles in other metabolic pathways (e.g., polyamine synthesis, transsulfuration, transmethylation, DNA synthesis) [165]. Overall, the biochemical cycle is complex and involves many metabolites and compounds. Green boxes and arrows demonstrate the resulting cellular functions. Symbols—Inhibits 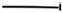 ; Stimulates/Activates 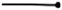 ; Enzyme Abbreviations—AHCY/SAH: S-adenosyl-L-homocysteine hydrolase; BHMT: Betaine-homocysteine S-methyltransferase; CBS: Cystathionine-β-synthase; CSE: Ɣ-cystathionase; DHFR: Dihydrofolate reductase; DNMTs: DNA methyltransferases; GCS: Ɣ-Glutamyl cysteine synthetase; GNMT: Glycine N-methyltransferase; GSS: Glutathione synthetase; HMTs: Histone methyltransferases; MAT: Methionine adenosyltransferase; MTHFR: 5,10-methylenetetrahydrofolate reductase; MTR: 5-methyltetrahydrofolate-homocysteine methyltransferase; MS: Methionine synthase; SAMDC: S-adenosylmethionine decarboxylase; SHMT: Serine hydroxymethyltransferase; TS: Thymidylate Synthetase.

Diabetes is one of the most severe metabolic disorders. It is caused by the lack of insulin or an insensitivity to insulin, which ultimately leads to high blood glucose levels and chronic damage in a wide range of organ systems. Among the diverse factors contributing to the pathogenesis of diabetes, epigenetic modifications have attracted attention because they can connect the pathology to environmental cues [141]. The methionine-homocysteine cycle, which generates methyl donors, is a major pathway important for DNA methylation (see Figure 4). The cysteine biosynthetic pathway is affected in diabetic individuals leading to higher levels of homocysteine in direct correlation with increased blood glucose levels [142]. Other intermediates of the methionine-homocysteine cycle are also altered in diabetes. These include folate, SAM, and *S*-adenosylhomocysteine [143,144,145]. Imbalance in the methyl donor levels can contribute to altered DNA methylation as well as histone methylation. The CpG-SNPs causing alterations in DNA methylation are also common in diabetes [125]. One example is the *IL2RA* gene promoter, which is regulated by DNA methylation [146]. Several genes implicated in the pathogenesis of diabetes are deregulated by altered DNA methylation. For example, decreased levels of *PPARGC1A* gene expression in diabetics are associated with increased DNA methylation at its promoter [147]. Examples of other diabetes-related genes regulated by DNA methylation include *GLP1R* [148], *PDX-1* [149], and *CTGF* [150]. Both human and mouse insulin genes are regulated by promoter methylation and MeCP2 binding [151]. Hyperinsulinemia is another metabolic condition associated with diabetes or mistakenly diagnosed as diabetes. Mice lacking MeCP2 expression develop insulin resistance and hyperinsulinemia [152]. Several girls with Rett syndrome, a neurological disorder primarily caused by mutations in the *MECP2* gene [44,109], have developed type 1 diabetes [153,154,155]. Because several diabetes-related genes are regulated by DNA methylation, it is highly likely that other MBPs might contribute to the pathogenesis of diabetes or related metabolic disorders.

#### 4.1.3. Imprinted Disorders

Imprinting disorders such as Prader-Willi syndrome (PWS)/Angelman syndrome (AS) (chromosome region 15q11-13), Beckwith-Weidemann syndrome, and Silver-Russell syndrome [156] are associated with a mutation or deletion of unique parent-of-origin DMRs in chromosome regions 15q11-13, 11p15.5, and 11p15 or maternal uniparental disomy of chromosome 7, respectively [84,157,158]. Changes in MBPs might also be associated with dysregulation of imprinted genes. Decreased MeCP2 protein expression (misregulated by miR-483-5p) and MeCP2 protein partners such as HDAC4 and TBL1X are observed in cells collected from Beckwith-Weidemann syndrome patients [159]. The imprinted locus *H19* is a *bona fide* target of MeCP2 [160,161]. Moreover, MeCP2 regulates other imprinted genes such as *UBE3A* [162,163] and *DLX5* [164]. Clinical overlap between Angelman and Rett syndromes highlight the potential involvement of MeCP2 in such imprinting disorders [87].

#### 4.1.4. Immune System-Related Disorders

The diverse types of terminally differentiated immune system cells types are generated through the process of differentiation of hematopoietic stem cells (HSCs). Hematopoietic stem cell differentiation requires a tightly controlled gene expression patterns, which is mostly acquired through epigenetic regulation [166,167,168] and many of the hematopoiesis-related genes are regulated by promoter methylation and examples include interleukins, CD5, CD29, PU1 and Pax5 [169]. Altered expression and mutations of DNMTs have been linked to impaired hematopoiesis, which can lead to immune disorders ranging from allergies to autoimmune disease [169]. Impaired immune system functions were reported in MBP-related disorders. For instance, patients and mice models with *MECP2* duplication syndrome show poor T-cell responses, high rate of infections, and altered levels of IFN-ɤ [170,171]. Polymorphisms of *MECP2* genes and MeCP2 overexpression were found to be a risk factor for systemic lupus erythematosus [172,173]. *MECP2* SNPs are also connected to the pathogenesis of primary Sjögren’s syndrome [174]. MBD2 is considered to be a candidate therapeutic target for autoimmune disorders [175], due to its presumed role in genome-wide DNA demethylation in systemic lupus erythematosus [176]. Similarly, MBD4 expression is elevated in systemic lupus erythematosus patients [177].

#### 4.1.5. Cardiovascular Diseases and Cerebral Ischemia

The impact of epigenetic factors on cardiovascular disease pathology has been shown in relation to DNA methylation and microRNAs. The potential of using these epigenetic marks as biomarkers for cardiovascular disease prediction has also been proposed [178]. In vascular disease conditions, global DNA hypomethylation and increased *S*-adenosylhomocysteine have been observed [179]. Moreover, hypomethylation of long interspersed nucleotide elements in blood samples were associated with an elevated risk of stroke and ischemic heart disease [180]. Several genes have been suggested to be misregulated by DNA methylation, which could lead to cardiovascular diseases. For example, gene-specific DNA hypomethylation of 15-lipoxygenase (*ALOX15*) and monocarboxylate transporter (*MCT3*) is associated with an increasing degree of atherosclerosis [178].

Cerebral ischemia or brain ischemia is a condition caused by a loss of blood supply to the brain, which leads to cerebral infarction, stroke, hemorrhage and death of brain tissue. In a mouse model of ischemic brain injury, increased DNA methylation and elevated methyl-group incorporation were observed. Moreover, loss of function of DNA methyltransferases lead to increased resistance to brain damage caused by ischemia. In the same mouse model, 5-Aza-mediated inhibition of DNA methylation suggested neuroprotection [181]. Similarly, reduced levels but not a complete loss of *Dnmt* in mouse post-mitotic neurons was associated with neuroprotection from ischemia [182]. Similar to cardiac ischemia, global hypomethylation represented by long interspersed nucleotide elements was linked to increased risk of cerebrovascular ischemia in males but not in females [183]. Therefore, together, these reports suggest a link between DNA methylation and cerebral ischemia and potential pharmacological applications of epigenetic modifiers such as DNA demethylating agents to confer protection from ischemia [184]. Illustrating the contribution of MeCP2 in brain ischemia, its protein levels were shown to be upregulated presumably through loss of miR-132 activity during ischemic preconditioning in mouse cortex [185].

### 4.2. Other DNA Binding Proteins Affected by DNA Methylation and Their Role in Human Diseases

Table 1 summarizes examples of other proteins, which bind to different methyl modifications. The evidence on the binding of specific proteins to methyl marks including corepressors such as SIN3A and NCOR2; coactivators such as CREB1; proteins which are involved in RNA splicing such as SRSF2/3, PRP31; DNA methyltransferases such as DNMT1; lysine methyltransferases such as EHMT1; and multifunctional epigenetic factors such as CTCF further demonstrate the diverse functions of DNA methylation multiple biological processes. Interestingly, the diverse nature of the regulatory proteins bound to 5caC (CTCF, EHMT1, NCOR2, PRP31 and DNMT1) hints the potential role of 5caC in chromatin organization, transcriptional regulation and RNA splicing (Table 1).

While the above mentioned proteins are bound to specific methyl modifications, binding to DNA for some of these proteins is prevented by other forms of methyl modifications found within their binding sites or adjacent to the binding sites. One protein belonging to this category and linked to many disease states is CTCF.

#### 4.2.1. CTCF

**Figure 5:**
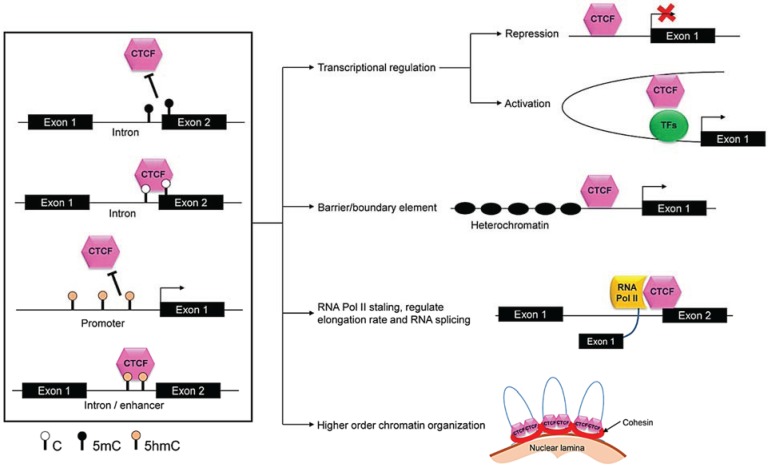
**Methylation-dependent CTCF functions.** CTCF binds to either unmethylated cytosine (C) or 5-methylcytosine (5mC) or 5-hydroxymethylcytosine (5hmC) depending on the genomic loci and the function. Once bound to DNA, it is involved in multiple functions including transcriptional regulation (repression and activation), barrier or boundary element, regulation of RNAPII-mediated transcriptional elongation and co-transcriptional splicing and modulation of higher order chromatin structure.

The recruitment of CTCF to intron/exon boundaries or to exons has shown to be ablated by 5mC methylation and shown to contribute to the regulation of DNA methylation-dependent alternative splicing [55]. However, it is still unclear whether the 5hmC methylation supports the binding of CTCF. There is controversial evidence with regard to the potential binding of CTCF to 5hmC. An enrichment of 5hmC was observed at the CTCF binding sites in enhancer regions within human ESCs [28]. In mouse spermatogonia, the enrichment of 5hmC at the promoter sequences bound by CTCF was lower while the CTCF-bound intronic sequences were enriched with 5hmC methylation [186]. In mouse ESCs, CTCF was shown to be bound to 5caC [114]. Therefore, it is possible that depending on the cell type or the genomic loci (enhancers, promoters, exons or introns), the binding of CTCF to methyl marks might be different, and so as the functions associated with its binding (see Figure 5).

CTCF was first reported as a transcriptional repressor for the chicken *C-Myc* gene [187]. Since then its functions have been expanded to multifunctional epigenetic modulator, which is involved in transcriptional activation and repression, barrier elements or boundary element (insulator), modulation of chromatin structure, and regulation of RNAPII-mediated transcriptional elongation and co-transcriptional splicing (see Figure 5). It has also been shown to be a critical factor in genomic imprinting through binding to the *IGF2/H19* imprinting control region [188]. Earlier it was thought that the multifunctional behavior of this single protein is determined by its interacting protein partners [189]. Some of the known protein partners of CTCF are key players of specific cellular processes such as higher order chromatin structure formation (cohesin) [190], transcription and elongation (RNAPII) [191], RNA splicing (RNAPII) [55] and XCI (YY1) [192]. On the other hand, considering the diverse nature of CTCF in binding to different methyl modifictions, it seems that the type of methyl modification is also a contributing factor to CTCF multifunctional behavior.

As a multifunctional epigenetic factor, its altered expression, binding to chromatin, and functions are associated with several human diseases. In breast cancer cells, both CTCF mutations [193] and altered expression of CTCF [194] are reported. Other than breast cancer, its role in cancer conditions was shown in many other cancer types such as prostate cancer [195], lung cancer [196], and colorectal cancer [197]. Due to its critical role in genomic imprinting, it is linked to imprinting disorders such as Beckwith-Wiedemann syndrome. In case of Beckwith-Wiedemann syndrome, demethylation of the *IGF2/H19* imprinting control region leads to the binding of CTCF and subsequently blocks the connection between an enhancer for *IGF2* and promotes activation of maternal *H19* [198]. CTCF is also involved in misregulation of other methylation-related epigenetic factors such as MeCP2. In autistic patient brains, the swapping of heterochromatin regions into the *MECP2* promoter and subsequent silencing is causing by increased promoter methylation and prevention of binding of CTCF [199].

## 5. DNA Methylation, Teratogens, and Neurodevelopmental Disorders

### 5.1. Normal Early Brain Development

During normal human embryo development, near the start of the third week of gestation, the midline and anterior-posterior axes are formed, guided by the movement of the mesodermal cells along the midline (primitive streak) in the caudal region. The primitive streak gives rise to the primitive pit, which merges with the mesodermal cells that form the notochord. The ectoderm dorsal to the notochord becomes the neural plate. The notochord initiates the differentiation of ectodermal cells into neuronal precursor cells via a gradient of signaling molecules, including retinoic acid (*i.e.*, vitamin A) and various peptide hormones: Wnt, FGF, TGF (BMP), sonic hedgehog (SHH) and Nodal. The neural plate, containing neural progenitor cells, elongates and starts to fold, forming a neural groove. The neural folds eventually meet and a tubular structure (the neural tube) forms by the start of the fourth week of gestation. By embryonic days 25 and 27, the anterior and posterior neuropores close, respectively. Neural crest cells, dorsal to the neural tube, migrate and differentiate into the sensory and sympathetic ganglia cells, adrenal neurosecretory cells and the enteric nervous system cells. The neural floorplate determines the polarity of the neural tube and subsequently gives rise to the motor neurons. The remaining ectodermal cells differentiate into the epidermis and the neural tube gives rise to the entire CNS [200,201,202].

Production of long projection neurons begins during the 6th week of gestation and finishes towards mid-gestation. Major structures of the brain, induced via various additional signaling molecules, are largely defined by the end of the embryonic period (8 weeks). During the early fetal period, extending to mid-gestation, the cortical neurons are generated. During the mid to late fetal period, maximal around mid-gestation and tailing off to roughly the 34th week of gestation, precursors of inhibitory interneurons and glial cells are produced. An exception is the cerebellum, where internal granular neurons are generated up to approximately 9 months after full term birth. Following generation, the cells migrate to their destined site and begin to differentiate. Differentiation begins in the fetal period but continues well after birth with the brain weight doubling in the first year of life, reaching 90% of adult weight by age six, and reaching full adult weight in early teen years [200,201].

Inadequate nutrition or the introduction of teratogens can affect normal embryonic or fetal brain development by altering the levels of signalling molecules and can lead to brain malformations. Early life experiences and the growth environment also influence postnatal brain development.

### 5.2. DNA Methylation and Brain Development

In mammals, DNA methylation reprogramming occurs soon after fertilization when the paternal and maternal genomes, packaged in their pronuclei, undergo demethylation. The paternal genome undergoes active demethylation before replication occurs, and the maternal genome undergoes passive demethylation, the exception being paternal and maternal imprinted genes. This is postulated to avoid the transfer of environmentally-acquired DNA methylation from the parents to the offspring. During implantation, for the zygote to obtain a totipotency status, widespread *de novo* remethylation occurs, creating distinct DNA methylation patterns in the blastocyst [203,204]. DNA methylation is orderly, termed the DNA methylation program (DMP). During neurulation, both 5mC and 5hmC are present in the neural tube and neural crest. In the mouse neuroepithelium during the proliferation and migration phases, 5mC appears first, followed by 5hmC and other active demethylation marks (5fC, 5caC). 5mC, MBD1, and DNMT1 appear in a high to low concentration gradient from ventral to dorsal, which coincides with the start of differentiation and maturation of neurons and neural crest cells. The appearance of 5hmC marks the differentiation of the neuroepithelium, coinciding with a loss of *Oct4* (a marker of undifferentiated cells) and the appearance of markers for neurogenesis (*Map2*) and proliferation (*Crabp1*). The DMP continues postnatally, with regional variations in 5mC/5hmC levels [205,206].

During early hippocampus development and neurogenesis (E15 to P7 in mice), each region of the hippocampus has its own DNA methylation program (DMP). Immature cells express 5mC while mature cells express 5hmC [207]. As the brain matures, a reduction in 5mC/5hmC coincides with chromatin compartmentalization; 5mC tends to be found in heterochromatin while 5hmC is found in euchromatin. The demethylation marks 5fC and 5caC are also present throughout mouse brain development from embryonic day 10 to postnatal day 45, but in much lower concentrations then 5mC and 5hmC [206].

Apart from CpG dinucleotide methylation, there is also CpH (H = A, T or C) methylation (non-CpG; see Section 2.1). Lister* et al.* [208] found evidence to support that CpH methylation is preferentially bound and driven by DNMT3A. It accumulates in neurons, but not in glia, in the postnatal (2 years after birth) and early adolescent human brain. Expression coincides with brain maturation and synaptogenesis, eventually becoming the main form of DNA methylation in human adult neurons of the frontal cortex. Both 5hmC and CpH methylation are found to be abundant in the rodent and human brain. During early postnatal mouse brain development, 5hmC levels rise and is restricted to CpGs. CpH levels increase in differentiating cells. Genes specific to neuronal and synaptic development are found to be CpH hypermethylated in glial cells (thereby suppressing expression) and both CpG and CpH hypomethylated in neurons. This suggests a potential role of CpH methylation in transcriptional repression of neuronal specific genes in the glial genome. Conversely, genes associated with oligodendrocyte function are CpH hypomethylated in glia and CpH hypermethylated in neurons. This further supports the idea that DNA methylation regulates brain maturation [208]. Guo *et al. 2014* [209] studied CpH methylation in the adult mouse dentate gyrus of the hippocampus in greater detail. *In vivo,* both CpH and CpG were found to be hypomethylated at neuronal transcription factor binding sites and transcription start sites, and regions of low CpG density contained CpH methylation. CpH methylation was also found to be more abundant in the adult brain than in the fetal brain, therefore supporting its role in post-natal brain maturation. MeCP2 and Dnmt3a were found to bind methylated CpHs* in vivo*. Both Dnmt1 and Dnmt3a are highly expressed in post-mitotic neurons. When CpH is demethylated, it can only be remethylated by *de novo* Dnmt3a [209].

Overall, DNA methylation and its binding partners, along with histone PTMs and chromatin organization drive early brain development, post-natal brain differentiation, and adult brain maturation. Aberrant DNA methylation can lead to impaired function and improper development. Furthermore, DNA methylation studies not only require CpG methylation analysis, but non-CpG methylation as well.

### 5.3. Effects of Teratogens on Normal Brain Development

Alterations to the cellular environment can be recorded in the epigenome. Adverse factors include teratogens such as recreational drugs (alcohol, tobacco, illicit drugs), toxins (lead, mercury), pollutants (BPA, PCB, PAH), poor nutrition (folic acid, choline, retinoic acid), maternal disease (diabetes), and even stress (through cortisol). Any of those factors could potentially alter the DNA methylome of the developing embryo or fetus. In terms of brain development, it could potentially lead to faulty organization of the nervous system or delayed neurogenesis. The most obvious and common effects on the newborn child are low birth weight and intrauterine growth restriction. Macroscopic congenital anomalies are the extreme. More subtle, but lifelong disabilities include diabetes, obesity, asthma, autism, depression, and fetal alcohol spectrum disorder (FASD) [205,210,211].

#### 5.3.1. Alcohol (Ethanol)

**Figure 6:**
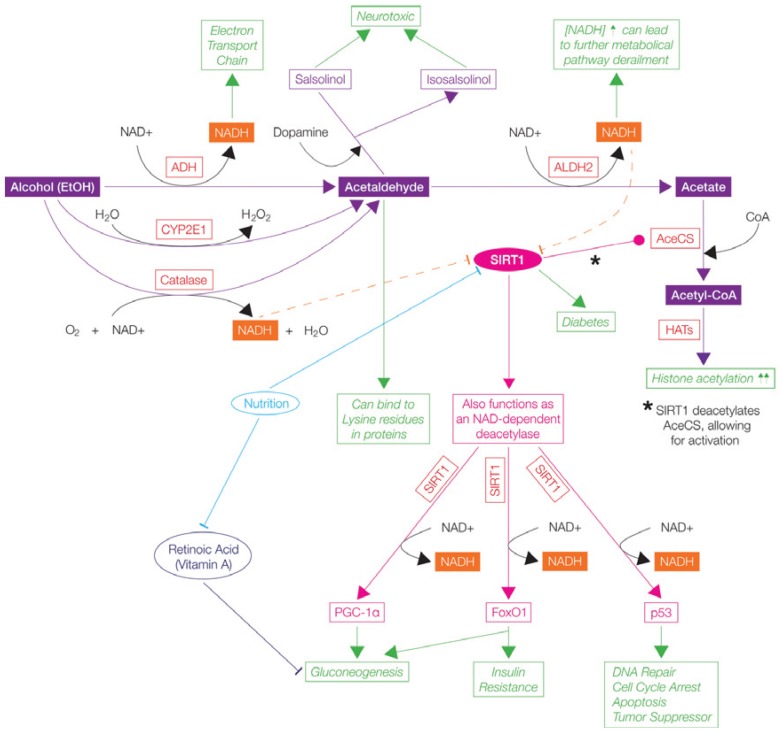
Alcohol (EtOH) metabolism and the effects its metabolites are exhibited in various metabolic and cellular processes. Various ethnic groups and populations have copy number differences and polymorphisms in the ADH gene, which in turn have an effect on how they metabolize alcohol [218]. High concentrations of alcohol (ethanol) modifies neurotransmitters (e.g., gamma amino butyric acid (GABA)_A_, *N*-methyl-D-aspartate (NMDA), NMDA-receptor (NMDAR) subunits) [165,212], while acetaldehyde can bind lysine residues in proteins as well as dopamine which results in salsolinol and isosalsolinol [212]. Although at first a stimulant, at higher quantities ethanol is also a depressant. This indicates that ethanol and its metabolites are essentially neurotoxic, and overtime can lead to a variety of health problems, including poor nutrition, addiction, mood disorders, cirrhosis of the liver and cancer [212,218,219]. Green boxes and arrows demonstrate the resulting cellular functions. Symbols—Inhibits. 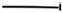; Stimulates/Activates 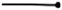; Enzyme Abbreviations-AceCS: Acetyl-CoA Synthetase; ADH: Alcohol Dehydrogenase; ALDH2: Acetaldehyde dehydrogenase 2; CoA: Coenzyme A; CYP2E1: Cytochrome P40 2E1; Fox01: Forkhead box protein 01; HATs: Histone Acetyltransferases; p53: tumor suppressor protein 53; PGC-1α: Peroxisome proliferation-activated receptor Ɣ coactivator 1α; NAD/NADH: Nicotinamide adenine dinucleotide; NADH: SIRT1: Sirtuin 1/Fast-activated longevity protein sirtuin 1 NAP-dependent deacetylase sirtuitin-1.

Alcohol is metabolized into acetaldehyde by alcohol dehydrogenase (ADH) in the liver, and by catalase and CYP3E1 in the brain. Acetaldehyde is further metabolized into acetate in all cells by acetaldehyde dehydrogenase (ALDH) (see Figure 6) [212]. During pregnancy, ethanol readily crosses the placenta, and is found at high concentrations in the amniotic fluid as well as in the fetal serum. Adverse consequences include growth restriction, congenital anomalies, and FASD [213].

The literature concerning epigenetic effects of alcohol is large. Ethanol is known to increase histone H3 acetylation, and increase or decrease H3 methylation levels. Ethanol can also alter SAM biosynthesis, which is a methyl donor for DNMTs and lysine methyltransferases, thereby having an effect on DNA methylation levels (generally hypomethylation). It also plays roles in other metabolic pathways (e.g., polyamine synthesis, transsulfuration, transmethylation, DNA synthesis) (see Figure 4) [165]. Both SAM and methionine are potential therapeutic targets for rescuing DNA methylation levels affected by alcohol and by other teratogens as well [165,214,215].

Liu* et al*. [216] studied the effects of alcohol on global DNA methylation in whole mouse embryos during neural tube formation using MeDIP-ChIP and Sequenom mass ARRAY EpiTYPER methylation detector. The alcohol-treated embryos showed growth restriction and developmental abnormalities in the heart, posterior neural tube, brain regions, and limbs. CpG island content (density profiles) were classified into three groups: high CpG (HCP) (mainly associated with housekeeping genes), intermediate CpG (ICP) and low CpG (LCP) (mainly associated with tissue specific and olfactory genes). HCP content accounted for more than two-thirds of the genome wide CpG content. Among the alcohol-treated embryos, those with closed neural tube had more alterations in methylation levels than did those with open neural tube (abnormal). Expression microarray showed that alcohol treatment caused alterations in methylation and expression of genes responsible for chromatin remodeling, neuronal morphogenesis, synaptic plasticity and neural development. Overall, these data suggest that alcohol strongly affects DNA methylation levels (both hypo- and hyper-methylation), which correlate with neural tube defects. The alcohol-effected gene promoters identified in this study were also found to be affected in nine other developmental syndromes that share phenotypic features with FASD [216].

Zhou* et al.* [217] performed a similar study using cultured adult rat dorsal root ganglion neural stem cells (DRG-NSC). Alcohol exposure delayed differentiation by changing the methylation status of genes necessary for that process (e.g*.*,* Igf2*,* Sox7*,* Cutl2*), and prevented the diversification of the DNA methylation (the programmed increase or decrease in DNA methylation of moderately methylated genes). The methylation patterns of the genes altered by alcohol were found to be related to neuronal receptors, neural development, synaptic transmission and olfactory (sensory) perception, all of which were similarly found to be affected by alcohol by Liu* et al.* [216] and were also validated by Sequenom mass ARRAY technology [217].

Laufer* et al.* [220] set out to study the possible mechanism behind gene expression changes in a mouse model of FASD by looking at DNA cytosine methylation, microRNAs (miRNAs) and CTCF binding sites in the adult mouse brain. Genome wide DNA methylation studies (MeDIP-ChIP and microarray with Ingenuity Pathway Analysis) revealed a large number (~1,000) of differentially methylated gene promoters in the alcohol-exposed mice. Most were genes related to cell development and maintenance, cell death, and brain development. Almost half of the genes known to be imprinted in the mouse genome showed changes in methylation. *In utero* alcohol exposure also had an effect on miRNA expression and the effect differed according to the timing of alcohol exposure (gestational day 8/11* vs.* gestational day 14/16). The affected miRNAs identified by an array were also found to be trimester dependent. Three of the imprinted regions with modified methylation have genes that are believed to be important in other neurodevelopmental diseases [220].

Most prenatal alcohol exposure studies focus on the mother, however, pre-conception alcohol habits in the father can also have an effect on DNA methylation. Bielawski* et al.* [221] treated adult male Sprague-Dawley rats with ethanol three times a week for 9 weeks and mated them with non-alcohol treated females. Sperm was extracted from alcohol treated males after mating and subjected to RNA extraction, followed by reverse transcription-polymerase chain reaction (RT-PCR) with primers for DNMT. It was concluded that the DNMT mRNA levels were significantly lower in the alcohol-treated male’s sperm when compared to the non-treated control males. This suggests that chronic alcohol consumption results in altered DNA methylation levels in sperm, which can have an effect on paternal genomic imprinting and can eventually be inherited by the offspring. However, one limitation to the study is that the authors did not distinguish between the different DNMTs [221].

Ouko* et al.* [222] studied the effects of paternal alcohol consumption on CpG methylation of imprinted genes *H19* and *IG* DMRs (discussed in Section 3.3) in male sperm. In normal males, both *H19* and *IG* DMRs are maternally expressed due to paternal *H19* and *IG* DMRs being more than 99% methylated in mature sperm. They are essential for normal neuro-behavioral development, with *H19* and *IGF2* being expressed at the same time. The *H19* DMR contains a CTCF binding domain that remains methylated, which in turn blocks CTCF binding. The *IG* DMR contains two genes (*DLK1* and *GTL2*), which play roles in the regulation of gene expression and in cellular differentiation. The authors postulated that paternal alcohol consumption could lead to hypomethylation of these DMRs via alcohol-induced demethylation and sought out to test their hypotheses in human males of mean age 25. Based on a survey questionnaire, men were classified into three categories: heavy, moderate, and non-drinkers. Semen (N = 16) was collected and subjected to DNA extraction, bisulfite modification and sequencing with primers for *H19* and *IG* DMRs. A total of 22 CpG sites were looked at for *H19* DMR and 10 CpG sites for *IG* DMR. Hypomethylation of both DMRs is seen in both drinking groups when compared to the non-drinking groups. For both DMRs, CpG site-specific hypomethylation was observed and enhanced in the drinking groups. Greater levels of demethylation were seen among the heavy drinkers than in the moderate drinkers, when compared to non-drinkers. With regards to the level of hypomethylation for both DMRs, *H19* DMR exhibited a greater decrease in methylation than did *IG* DMR, indicating that *H19* is more sensitive to alcohol-related demethylation [222].

Stouder* et al.* [223] studied transgenerational effects in mice exposed to low dose alcohol *in utero*. The methylation pattern of two paternally imprinted genes (*H19* and *Gtl2*) and three maternally imprinted genes (*Peg1, Snrpn and Peg3*) were assessed in various tissues (including sperm) across two generations. Among the five genes selected, only *H19* methylation was affected [223]. As seen in Ouko* et al.* [222], hypomethylation of *H19* CpGs was detected, with specific CpG sites within CTCF 2 binding site showing high decreases in methylation [223]. Knezowich* et al.* reported a similar reduction in methylation for *H19* in male alcohol exposed mouse sperm [224]. Among the F2 male offspring, no change in *H19* CpG methylation levels was noted in somatic tissues, which does not correlate with Knezowich* et al.’s* findings. However, in the F2 newborn whole brain (but not hippocampus) a decrease in *H19* CpG methylation was seen [223]. These studies overall show the potential use of *H19* CpG methylation as a biomarker for paternal alcohol exposure.

For more details regarding imprinted genes, including *H19*, in the brain, please refer to a review by Kernohan and Bérubé’s [84].

#### 5.3.2. Tobacco (Nicotine)

Maternal tobacco smoking exposes the developing fetus to over 4,000 toxic compounds of which ~30 have been linked to adverse health consequences. Nicotine is known to cross the placenta and is found in higher concentrations in fetal serum than in maternal serum. Nicotine exposure has an effect on the growth of the fetus through vasoconstriction, which reduces blood flow in the placenta and umbilical cord. Nicotine exposure also has an effect on brain development [213].

Maccani* et al.* [225] studied the effects of maternal smoking on CpG methylation in human placentas. They hypothesized that maternal smoking during pregnancy was associated with CpG DNA methylation changes in the placenta, and that these changes were associated with preterm birth (<37 weeks gestation). Placentas sampled 2 cm from the umbilical cord insertion site of smokers (*n* = 22) and non-smokers (184). The authors found that 1918 CpG loci and their methylation patterns were associated with maternal smoking. They highlighted seven CpG loci that resided in the *RUNX3* gene, which codes for a transcription factor, is said to function as a tumor suppressor, and is known to be associated with Asthma and airway hyper-responsiveness. Pyrosequencing confirmed hypermethylation of two of the seven CpG loci highlighted (cg04757093 and cg00117172), and statistical analyses showed an association between two of the seven loci with preterm birth (cg04757093 and cg14182690). Therefore, the authors concluded that hypermethylation of CpG loci cg04757093 was significantly associated with maternal smoking during pregnancy and preterm birth. However, the exact role of *RUNX3* in the placenta is unknown. A few limitations to this study include a small sample size (N = 22), unknown dosage of exposure, and the sensitivity of the DNA methylation profiling technique used [225].

Joubert* et al.* [226] performed a similar study, examining CpG methylation patterns in 1,062 newborns born to smoking mothers (MoBa cohort). The association between a nicotine metabolite cotinine in the mothers’ blood and CpG methylation in the newborns’ cord blood was analyzed. The authors identified 26 CpGs mapped to 10 genes that were significantly associated with maternal plasma cotinine and were found to be differentially methylated. The genes with the most CpGs identified were *GFI1* with eight CpGs, and *AHRR*, *MYO1G* & *CYP1A1*, all with four CpGs each. The results were replicated in another study sample size (N = 18) (NEST cohort). The most significant finding in both cohorts was an increase in cotinine levels (or reported maternal smoking), which was associated with a decrease in CpG methylation at cg05575921 in the *AHRR* gene. *AHRR* codes for a repressor protein involved in the aryl hydrocarbon receptor-signaling cascade, which mediates dioxin toxicity. It is also said to be involved in cell growth and differentiation [226].

Li *et al*. [227] looked at the effects of nicotine *in utero* and how it may have had an effect on the offspring’s susceptibility to hypoxic-ischemic encephalopathy (HIE) as well as on Angiotensin II (AngII) receptors AT_1_R and AT_2_R. AngII, through its receptors, helps regulate the cardiovascular system as well as physiological responses. If affected, it can lead to cardiovascular disease, hypertension, diabetes, and stroke. Pregnant rats were given nicotine injections throughout the pregnancy, and offspring at fetal day 21 (F21/E21) and post-natal day 10 (P10) were sacrificed for brain removal. Prenatal nicotine exposure was found to have negative effects on growth (compared body and brain size) in both F21 and P10 offspring when compared to saline controls and to increase the brain’s vulnerability to HI-induced infarct. In P10 prenatal nicotine exposed pups, CpG methylation analyses at the AT_2_R promoter revealed significant hypermethylation upstream of a TATA-box in males but not in females. This suggests prenatal nicotine exposure alters sex-specific AngII receptors CpG methylation in the developing brain, more so AT_2_R then AT_1_R, which in turn, affects their expression levels. AT_2_R reduction increases susceptibility to hypoxic-ischemic brain damage, which could explain some of the adverse effects of prenatal nicotine exposure [227].

Breton* et al.* [228] studied global and promoter CpG island methylation in buccal cells of 5–6 year old children. Tobacco smoke exposed children had hypomethylation of *AluYb8* (a repeated DNA sequence found in >2,000 loci in the human genome), with no significant difference for *LINE1* methylation. Pyrosequencing showed gene promoters with the most significant differences were *AXL* (a receptor tyrosine kinase) and *PTPRO* (a receptor protein tyrosine phosphatase) bisulfite [228].

Toledo-Rodriguez* et al.* [229] obtained genomic DNA from blood leukocytes of adolescents whose mothers had smoked during pregnancy and used bisulfite sequencing to study brain derived neurotrophic factor (BDNF), the gene product of which is an important regulator of brain cell growth. They demonstrated increased amounts of DNA methylation in exon 6 of *BDNF* gene. The authors speculate that cigarette smoking during pregnancy could have an adverse effect on brain development [229]. However, the authors did not control for sex [229]. This was rather disappointing, since it is well documented that sex differences exist not only in the brain (e.g., males are larger than females; morphological and neurochemical differences), but also at an epigenetic level (e.g., in neonatal brain, males have higher H3 acetylation than females) [230,231]. However, it must be noted that such human studies are complicated by the likelihood that pregnant women who smoke tobacco also tend to smoke in the home after the child is born.

For further details about the effects of maternal nicotine on human brain development, please see Morris’s* et al. 2011* review, and for human and rodent brain please see Pagani’s* et al.* 2013 review [231,232].

#### 5.3.3 Cocaine

Cocaine easily crosses both the placenta and the blood-brain barrier, thereby having direct effects on the fetal brain [213]. Novikova* et al.* [233] exposed pregnant CD1 mice to cocaine twice daily, sacrificed the pups at postnatal days 3 and 30, harvested the brains, and studied global and CpG island DNA methylation levels as well as *Dnmt* gene expression levels. A 30% decrease in global DNA methylation levels was found in the Day 3 cocaine-exposed offspring. The CpG islands affected by cocaine were found to be 34% hypermethylated (five neural genes, 24 housekeeping genes) and 66% hypomethylated (seven neural genes, 57 housekeeping genes). Bisulfite sequencing was used to specifically identify CpG islands associated with hypomethylated and hypermethylated gene promoters (see reference for full list). Among the P30 cocaine exposed offspring, there was a 35% decrease in global DNA methylation levels and an increase in both *Dnmt1* and *Dnmt3a* expression levels. Approximately 67% of the CpG islands showed stable alteration when the two ages were compared leading the authors to conclude that maternal cocaine exposure persistently alters DNA methylation in the offspring [233].

Anier* et al.* [234] also looked at DNA methylation machinery (Dnmt, Mecp2) in the *nucleus accumbens* (NAc) and hippocampus of the adult mice exposed to acute and repeated cocaine treatments. Acute cocaine treatment had no effect on *Dnmt1* mRNA levels, however there was an upregulation of *Dnmt3a/b*. This led to the hypothesis that an upregulation of Dnmt3a/b would result in hypermethylation of certain CpG island associated gene promoters that have been found to be associated with cocaine (*Bdnf*,* PP1c*,* FosB* and *A_2A_R*). *PP1c* was found to be hypermethylated at the CpG island associated promoter region 24 h after acute and repeated cocaine treatment and MeCP2 binding to *PP1c* promoter increased. *FosB* was found to be hypomethylated at the CpG island associated promoter region 1.5 h after acute and repeated cocaine treatment and MeCP2 binding at *FosB* promoter decreased. Therefore, cocaine treatment in the adult mouse NAc results in alterations in gene expression by changing DNA methylation levels [234].

Tian* et al.* [214] studied global DNA methylation levels in adult mouse brains after cocaine exposure using liquid chromatography-electrospray ionization tandem mass spectrometry (LC-ESI/MS/MS) [214]. The LC-ESI/MS/MS method is a highly sensitive method for distinguishing 5mdC (deoxyribonucleoside) from 5mC (ribonucleoside) [235]. Global DNA methylation levels decreased in the prefrontal cortex but not *nucleus accumbens* in the cocaine group. They also looked at components of the DNA methylation machinery (Dnmt3a/b and MeCP2) at the mRNA and protein level by RT-PCR and Western Blot respectively. Both mRNA and protein expression levels of *Dnmt3b* were downregulated, and could be rescued by treatment with methionine, a methyl donor [235].

#### 5.3.4. Other Drugs

One of marijuana’s main psychoactive compounds, tetrahydrocannabinol (THC), rapidly crosses the placenta and is concentrated in the fetus for up to 30 days, thereby prolonging gestational exposure [213]. To our knowledge, there have been no papers published on the effects of *in utero* exposure to cannabis (THC) on DNA methylation and the resulting effects on the offspring. However, others have studied the effects of marijuana on dopamine receptor D (DRD2) in human fetal brains as well as in rat post-natal (comparable to human fetal brains) and adult brains [37]. For further details about the effects of maternal cannabis on brain development, please see Morris’* et al.* 2011 review [32].

Like cocaine, methamphetamine (METH) readily crosses both the blood-brain barrier and the placenta, thereby having direct effects on the developing brain [213]. Itzhak* et al.* [236] took adolescent female and male mice, exposed them to METH into adulthood and the mating period with continued exposure during gestation. Hippocampi of the offspring underwent DNA methylation analysis using MeDIP-chip and bisulfite sequencing. METH caused DNA hypermethylation in promoters of genes essential to synaptic plasticity, learning, and memory. Enrichment analysis with histone PTM libraries also showed hypermethylation in promoters that possess active histone PTMs (H3K4me3) and demethylation in promoters that possess silencing histone PTMs (H3K27me3) [236].

Semi-synthetic opioids (heroin), which are metabolized into opiates (morphine and codeine), rapidly cross the placenta, with levels of opiates equalizing between the mother and fetus [213]. Using the high-performance liquid chromatography with ultraviolet detection (HPLC-UV) method, Fragou* et al.* found no significant change in global DNA methylation rodent brains exposed to heroin or cocaine [237]. It is not clear why the cocaine result differs from the experiment by Itzhak, but could be due to improper hydrolysis of DNA or perhaps RNA contamination during DNA isolation [235]. MeDIP-chip with bisulfite sequencing is now considered the more appropriate method for DNA methylation studies [238].

### 5.4. MeCP2 in Neurodevelopmental/Neurological Disorders

Among all the MBPs, MeCP2 is considered as the best studied example for involvement of epigenetics in neurological disorders [239,240]. The role of MeCP2 in multiple cellular processes and its relation to human diseases were discussed above. Even though MeCP2 is ubiquitously expressed, its highest expression is reported in brain [241], and the majority of the MeCP2-deficient or MeCP2 overexpression disorders show neurological phenotypes. Within the brain, MeCP2 expression is detected in many cell types including neurons, astrocytes, and oligodendrocytes [242,243,244]; alterations in the expression within all these cell types have been linked to the development of severe neurological complications [109]. The MeCP2 associated brain disorders can be broadly categorized as neurodevelopmental and neurodegenerative disorders. These disorders can be caused by or associated with loss of function mutations of *MECP2*, altered expression (either upregulation or downregulation), and polymorphisms or sequence variants. Examples of MeCP2-associated disorders are discussed in detail below.

#### 5.4.1. Rett Syndrome

Mutations causing loss of function or loss of expression of *MECP2* are the primary cause of Rett syndrome [245,246]. The majority of Rett syndrome-causing mutations are clustered in the MBD and TRD domains, while some mutations are also found in the C-terminus and N-terminus. These mutations have been shown to cause loss of binding to chromatin, loss of interactions with the protein partners including the complexes for transcriptional repression, loss of transcription repression activity, and loss of MeCP2 PTMs (phosphorylation) required for neuronal activity-dependent target gene expression regulation [109]. The latest studies on MeCP2 mutations show that the major cause of Rett syndrome is mediated through the loss of the DNA-MeCP2 bridge [247,248]. Known *MECP2* mutations can abolish MeCP2 binding to 5mC or 5hmC [109]. Therefore, it is evident that the loss of interaction with the methylated DNA and methylation-dependent functions of MeCP2 are contributing to the pathogenesis of Rett syndrome.

#### 5.4.2. Autism Spectrum Disorders

Illustrating the role of MeCP2 as well as DNA methylation in autism, increased promoter methylation and loss of the binding of CTCF were shown to be correlated with significantly reduced MeCP2 expression in autistic brains [199,249]. MeCP2 overexpression has also been shown to be a cause of autism [250]. Apart from the altered MeCP2 expression, other variations to MeCP2 were also reported in autistic patients. Both loss of function *MECP2* mutations [251,252,253,254] and sequence variants are found to be associated with this devastating disease [255,256]. Even though *MECP2* duplication syndrome is a different disorder, it shares some of the autistic phenotypes [257,258]. MeCP2 has been linked to autism spectrum disorders through its target genes such as *RELN* and *UBE3A* which are epigenetically regulated and are misregulated in autism spectrum disorders [88].

#### 5.4.3. Fetal Alcohol Spectrum Disorders

Prenatal exposure of the embryo to alcohol during pregnancy leads to a spectrum of neurological complications, which are collectively referred to as FASD. The role of DNA methylation is the pathogenesis of FASD and ethanol teratogenesis was discussed in Section 5.3. The first link between FASD and MeCP2 was reported in a patient with a *MECP2* mutation in AT-Hook domain within the TRD, and this girl showed phenotypes of both Rett Syndrome and FASD [259]. The AT-Hook domain of MeCP2 is known to mediate MeCP2 binding to DNA [260]. Since then, the potential involvement of MeCP2 in FASD pathogenesis has been studied in many* in vivo* and* in vitro* FASD models [207,261,262,263,264]. Alterations in expression and distribution of MeCP2 have been reported in these FASD models in response to ethanol exposure. Ethanol seems to alter the expression of MeCP2 in a cell type-specific as well as brain region-specific manner. For example, ethanol downregulated MeCP2 in the cortex and striatum of ethanol exposed offspring [261,265]. Reduced MeCP2 levels were also reported in ethanol exposure hypothalamus, which was corrected through choline treatments [266]. In contrast, in the ethanol exposed hippocampus, ethanol upregulated MeCP2 significantly [264,267]. In the ethanol exposure mouse dentate gyrus, the expression of MeCP2 was reduced and distribution of MeCP2 was altered [207].

#### 5.4.4. Other

Apart from the above-discussed neurological conditions, MeCP2 is found in many neurological, neurodevelopmental, and neurodegenerative disorders. Alzheimer’s disease is an example for the association of MeCP2 with neurodegenerative disorders. MeCP2 can contribute the pathogenesis of Alzheimer’s disease through sequence variants of *MECP2* [268] as well as through regulation of its target gene *BDNF*, which is a hot gene in Alzheimer’s disease [268,269]. Mental retardation is a major phenotype seen in many MeCP2-related disorders including Rett Syndrome and autism. *MECP2* mutations are also found in X-linked mental retardation [270] and AS [271]. Reduced levels of MeCP2 expression were also reported in cases of Down Syndrome and PWS [249].

## 6. DNA Methylation as a Biomarker for Human Diseases

A biomarker is defined as a measurable indicator of a biological state or disease. DNA methylation can be considered as a hallmark of many human diseases and, thus, can be utilized as potential biomarker. The application of DNA methylation to identify diseases has already been reported in diseases such as diabetes, neurological disorders, cardiovascular disorders and is extensively applied in cases of cancer [272]. Utilization of DNA methylation is advantageous compared to the expression-based biomarkers due to few reasons including easy PCR-based amplification using fewer cell numbers and can be detected in samples collected by non-invasive methods (saliva, blood, urine, semen) [272]. However, differential methylation profiles in different cell types make it challenging to use it as a biomarker, since heterogeneity of the sample might provide biased or inaccurate results. In order to address this issue, several bioinformatics tools have been developed to deconvolute the methylation data obtained from a heterogeneous population into individual cell types [273,274].

### 6.1. Type 2 Diabetes and the DNA Methylome

Type 2 diabetes mellitus (T2DM), the most common form of diabetes, has become a major concern worldwide. A recent study in England has reported that the percent of the population with pre-diabetes has dramatically increased over the past few years [275]. Similar trends are observed worldwide. In Canada, there are more than 60,000 new cases of T2DM yearly. Lifestyle, diet, environment, and genetics contribute to the onset of T2DM. Genome-wide association studies have identified T2DM risk genes, which have roles in pancreatic beta-cell mass and/or function [276]. Several studies have reported that epigenetic changes are also involved, with an alteration in the DNA methylation patterns in the beta cells of the pancreas and white blood cells of patients with type 2 diabetes being [277,278].

The first detailed DNA methylation profiling in the pancreatic islets of T2DM patients (five) and non-T2DM (eleven) healthy donors was reported by Volkman, M* et al.* [276]. Using the Illumina Infinium HumanMethylation27 BeadChip array, Francois Fuks’ team identified 276 CpG loci associated with the promoters of 254 genes that had differential DNA methylation in the diabetic islets, with the majority (96%) showing decreased methylation in the T2DM sample [276]. The differentially methylated genes have roles in beta-cell function, cell death, and adaptation to metabolic stress. Global changes in DNA methylation were not observed; rather the changes were gene specific. Importantly, these differentially methylated CpGs did not overlap with known SNPs. Several of these differentially methylated sites were validated by bisulfite pyrosequencing. In contrast to pancreatic beta-cells, minimal alterations in methylation patterns were observed in the peripheral blood leukocytes from T2DM patients and controls.

The reduced methylation of the upstream promoter region CpG in the T2DM beta-cells often correlated with increased expression of the associated gene; however, as noted by the authors, there is not a simple relationship between reduced promoter DNA methylation and increased transcriptional activity. Further analyses of the differentially methylated upstream promoter regions identified the GATA transcription factor-binding motif.

In a more recent analysis of the DNA methylome of pancreatic beta cells from T2DM patients, Dayeh T and colleagues used the Illumina Infinium HumanMethylation450 BeadChip [277]. As with the Fuks’ study, global changes in DNA methylation were not observed (pancreatic islets from fifteen T2DM and thirty-four non-diabetic donors). The authors identified 1,649 CpG differentially methylated sites, with 1,008 located in or near 853 unique genes, some of which have functions in pancreatic islets, exocytosis, and apoptosis, and 561 being intergenic. Of the 276 differentially methylated CpG sites identified in Fuks’ study, 71 sites were found to be differentially methylated in this more recent study. The majority (97%) of the differentially methylated sites were methylated at a lower level in the T2DM islets. Chromosomes 1 and 2 had more differentially methylated sites than the other chromosomes, with the differentially methylated sites being underrepresented on Chromosome 19. Of the 853 differentially methylated genes, 102 genes were differentially expressed in T2DM compared to non-diabetic islets. As with the Fuks’ study, the majority (75%) of these genes had decreased methylation and increased expression in the T2DM islets. The differentially methylated genes have roles in cancer, axon guidance, MAPK signaling, focal adhesion, and actin cytoskeleton. An overrepresentation of CpG sites in the 5ꞌ UTR was observed with the genes showing this inverse relationship. Interestingly there was an overrepresentation of differentially methylated CpG sites in the gene body; possibly these differentially methylated sites could alter splicing of the transcripts produced by these genes in T2DM relative to non-diabetic islets.

Differential methylation was observed for 17 T2DM candidate/risk genes, including *IRS1*, *FTO*, and *TCF7L2*. The authors proposed that disease susceptibility might be influenced by a combination of epigenetic and genetic events. In a previous study, these authors showed that around 50% of the SNPs associated with T2DM were CpG-SNPs, which could delete or introduce methylated sites; this observation highlights the importance of knowing whether a SNP is responsible for a change in the methylation status of a CpG [125,279].

In contrast to the results reported by the Fuks’ study, Toperoff* et al.* identified DNA methylation variations that served as an early marker of T2DM in peripheral blood leukocytes [278]. The authors used a two-step approach, first doing a comparison of pooled DNA from T2DM with that from non-diabetic controls, which identified differentially methylated regions followed by a deep sequencing approach to explore specific regions of interest. DNA from peripheral white blood cells of pooled T2DM patients (710) and non-diabetic control (459) were analyzed by digestion with methylation-sensitive restriction enzymes followed by hybridization to Affymetrix SNP6 microarrays. Linkage disequilibrium blocks containing T2DM-associated sequence polymorphisms were enriched in the differentially methylated regions. Further analyses of these regions by bisulfite conversion and deep sequencing identified differentially methylated CpGs. One of these differentially methylated sites was found in the intron of the *FTO* gene, which has a T2DM-associated polymorphism (A is the risk allele* versus* G) position 11 bp upstream from the differentially methylated CpG. The risk allele was found to be significantly hypermethylated compared to the other allele [278,279]. However, this risk allele was hypomethylated in DNA from T2DM patients.

Interestingly the DMRs were often co-localized with enhancers and binding sites of methylation sensitive transcription factors such as USF1/2, MYCN and E2F. The USF1/2 transcription factor has a role in controlling glucose-lipid metabolism in response to insulin and is involved in beta-cell development.

Together the studies suggest that DMRs in the islets may play a role in the pathogenesis of T2DM. Some of these sites may predispose the individual to T2DM, while others may be a consequence of altered gene expression in the T2DM islets. For those differentially methylated sites found in peripheral blood leukocytes, particularly those that alter transcription factor binding to enhancer regions, these sites may predispose the individual to T2DM. It will be interesting to determine whether these sites are inherited and represent a transgenerational epigenetic modification.

### 6.2. DNA Methylation as a Biomarker for Neurological Disorders

The neurological disorders that show disease-specific DNA methylation patterns and/or changes in methylation-related genes were discussed in previous sections. In such cases the utilization of DNA methylation and/or related proteins as a biomarker could be achievable [280,281]. In order to study the application of DNA methylation as a biomarker, twin studies serve as powerful tools. Despite their mostly identical genetic basis, they might display changes in DNA methylation. Easy access to the cells or tissue for biomarker analysis is one critical requirement for a good biomarker. Except for brain tumors or in autopsy studies, obtaining brain samples for biomarker analysis is challenging and in most cases is not realistic. Therefore, blood-based DNA methylation was suggested as a non-invasive biomarker surrogate for neurological disorders [281]. In light of this, DNA methylation immunoprecipitation for the DMRs on chromosome 21 was successfully used to detect fetal trisomy 21 in maternal peripheral blood samples [282]. Since some neurological and psychiatric disorders (including depression, schizophrenia, and Parkinson disease) also demonstrate disease-specific methylation patterns, it was suggested that blood-based non-invasive biomarker analysis can be applied in these situations [281]. Moreover, since *MECP2* mutations are the primary cause of Rett syndrome, it is already used as a biomarker to detect Rett syndrome. However, such biomarkers must be utilized carefully, because neurological disorders are highly heterogeneous. For instance, there are Rett syndrome cases which lack *MECP2* mutations, while presence of *MECP2* mutations are reported in non-Rett syndrome cases [109]. Hence, utilization of an epigenetic factor like DNA methylation, which is susceptible to environmental changes, should be carried out with caution and in concert with other analysis to confirm the diagnosis.

Moreover, placental expression of many imprinted genes (examples: *IGF2*, *MEG3*, *PEG3*) which are known to be regulated by DNA methylation-mediated epigenetic mechanisms was associated with infant neurodevelopmental outcomes [283]. In a another study, it was shown that increased promoter methylation of 11-beta hydroxysteroid dehydrogenase gene (*HSD11B2*) was associated with lower birth weight and lower quality of movement scores, which are considered as predictors of early/infant neurobehavioral outcomes [284]. These examples further strengthen the notion that DNA methylation could be used as biomarkers for neurological disorders. Studies also suggest that not only DNA methylation, but also other epigenetic modification could presumably be used as biomarkers for human diseases. One such epigenetic mechanism is microRNAs, the expression of which has been linked to the early neurobehavioral outcomes. Placental expression of several microRNAs including miR-16, miR-146a, and miR-182 was correlated with neurobehavioral outcomes such as attention scores and movement score [285].

## Abbreviations

5caC: 5-carboxylcytosine
5fC: 5-formylcytosine
5hmC: 5-hydroxymethylcytosine
5mC: 5-methylcytosine
ALDH: acetaldehyde dehydrogenase
ADH: alcohol dehydrogenase
AS: Angelman syndrome
APEX1: AP endonuclease 1
AP site: apurine/apyramidine site
BER: base excision repair
BRM: bromodomain
CREB1: cAMP-response element-binding protein-1
CEBPA: CCAAT/enhancer binding protein alpha
CTCF: CCCTC-binding factor
CNS: central nervous system
ChIP: Chromatin immunoprecipitation
ChIA-PET: chromatin interaction analysis by paired-end-tag sequencing
CTD: C-terminal domain
CXXC: Cys-X-X-Cys chromatin-associated binding domain
DMRs: Differentially methylated regions
DNMTs: DNA methyltransferases
DNMT1: DNA-methyltransferase 1
E: Embryonic day
ESCs: Embryonic Stem cells
EHMT1: Euchromatic histone-lysine N-methyltransferase 1
EZH2: enhancer of zeste homoloque 2
FASD: Fetal Alcohol Spectrum Disorders
HCC: hepatocellular carcinoma
HP1: heterochromatin protein 1
HDAC: histone deacetylases
SETDB1: histone-lysine N-methyltransferase
ICR: imprint control region
ID: inter domain
KAT: lysine acetyltransferases
MBD: Methyl Binding Domain
MeCP2: Methyl CpG Binding Protein 2
miRNAs: microRNAs
ncRNAs: noncoding RNAs
NCOR2: Nuclear receptor co-repressor 2
NuRD: nucleosome remodeling deacetylase
SIN3A: Paired amphipathic helix protein Sin3 transcription regulator homolog A
PHD: plant homeodomain
PRC2: polycomb repressive complex 2
PWS: Prader-Willi syndrome
PGC: primordial germ cells
PAR: pseudo-autosomal region
RNA: ribonucleic acid
SAM: *S*-adenosyl methionine
SRSF: Serine/arginine-rich splicing factor
SNPs: single nucleotide polymorphisms
TET: ten-eleven translocation
TDG: thymine DNA glycosylase
TRD: transcription repression domain
T2DM: Type 2 diabetes mellitus
PRP31: U4/U6 small nuclear ribonucleoprotein
UHRF1: Ubiquitin-like PHD and RING finger domains 1
UTR: untranslated regions
USC: uterine serous carcinoma
XIC: X inactivation center
XCI: X-chromosome inactivation
FGF: Fibroblast growth factor
TGF/BMP: Transforming growth factor (Bone morphogenetic portines)
SHH: sonic hedgehog
Oct4: octamer-binding transcription factor 4
Map2: Microtubule-associated protein 2
Crabp1: Cellular retinoic acid-binding protein 1
Sox2: SRY/Sex determining region Y-box 2
Nanog: Homeobox protein NANOG
GABAA: γ-aminobutyric acid receptor
NDMA: *N*-methyl-D-aspartate
NMDAR: *N*-methyl-D-aspartate Receptor
HATs: Histone Acetyltransferases
Igf2: Insulin-like Growth Factor 2
Sox7: SRY/Sex determining region Y-box 7
Cutl2: Cut-like homeodomain transcription factor 2
Gria3: glutamate receptor ionotropic AMPA 3
Adrala: Adrenergic receptor
H19: imprinted maternally expressed non-coding RNA
IG: DLK1-GTL2/MEG3 intergenic region
Gtl2: Maternally expressed 3 non-coding RNA
Dlk1: protein delta homolog 1 transmembrane protein
Peg1: Mesoderm-specific transcript homolog protein (MEST)
Snrpn: Small nuclear ribonucleoprotein-associated protein N
Peg3: imprinted Paternally-expressed gene 3 protein
AluYb8: short interspersed nucleotide element
PTPRO: protein tyrosine phosphatase, receptor type, O
LINE1: long interspersed nucleotide element
AXL: AXL receptor tyrosine kinase
BDNF: brain derived neurotrophic factor
HIE: hypoxic-ischemic encephalopathy
AngII: Angiotensin II
AT_1_R, AT_2_R: AngII type 1 and type 2 receptors
PP1c: protein phosphatase-1 catalytic subunit
A2AR: Adenosine Receptor 2
fosB: FBJ murine osteosarcoma viral oncogene homolog B
DRD2: dopamine receptor D
DMP: DNA methylation program
PTMs: Post translational modifications
SNPs: small nucleotide polymorphisms
METH: methamphetamine
NAc: nucleus accumbens
THC: Tetrahydrocannabinol
RUNX3: Runt-related transcription factor 3
GFI1: Growth factor independent 1
AHRR: Aryl-hydrocarbon receptor repressor
MYO1G: Myosin 1G
CYP1A1: Cytochrome P450 1A

## References

[1] Dahm R. (2005). Friedrich Miescher and the discovery of DNA. Dev. Biol..

[2] National Center for Biotechnology Information Pubmed Search “DNA”. http://www.ncbi.nlm.nih.gov/pubmed/?term=“DNA”.

[3] National Human Genome Research Institute (NHGRI) The Human Genome Project. http://www.genome.gov/25019879.

[4] Hallgrimsson B., Hall B.K. (2011). Epigenetics: Linking Genotype and Phenotype in Development and Evolution.

[5] Razin A., Cedar H. (1977). Distribution of 5-methylcytosine in chromatin. Proc. Natl. Acad. Sci. USA.

[6] Pollack Y., Stein R., Razin A., Cedar H. (1980). Methylation of foreign DNA sequences in eukaryotic cells. Proc. Natl. Acad. Sci. USA.

[7] Razin A., Riggs A. (1980). DNA methylation and gene function. Science.

[8] National Center for Biotechnology Information Pubmed Search “DNA Methylation”. http://www.ncbi.nlm.nih.gov/pubmed/?term=“DNA+methylation”.

[9] Hashimoto H., Vertino P.M., Cheng X. (2010). Molecular coupling of DNA methylation and histone methylation. Epigenomics.

[10] Schäfer A., Karaulanov E., Stapf U., Döderlein G., Niehrs C. (2013). Ing1 functions in DNA demethylation by directing Gadd45a to H3K4me3. Genes. Dev..

[11] Bian C., Xu C., Ruan J., Lee K.K., Burke T.L., Tempel W., Barsyte D., Li J., Wu M., Zhou B.O. (2011). Sgf29 binds histone H3K4me2/3 and is required for SAGA complex recruitment and histone H3 acetylation. EMBO J..

[12] Schram A.W., Baas R., Jansen P.W., Riss A., Tora L., Vermeulen M., Timmers H.T. (2013). A dual role for SAGA-associated factor 29 (SGF29) in ER stress survival by coordination of both histone H3 acetylation and histone H3 lysine-4 trimethylation. PLoS One.

[13] Ndlovu M.N., Denis H., Fuks F. (2011). Exposing the DNA methylome iceberg. Trends Biochem. Sci..

[14] Laurent L., Wong E., Li G., Huynh T., Tsirigos A., Ong C.T., Low H.M., Kin Sung K.W., Rigoutsos I., Loring J., Wei C.-L. (2010). Dynamic changes in the human methylome during differentiation. Genome Res..

[15] Guo J.U., Su Y., Zhong C., Ming G., Song H. (2011). Hydroxylation of 5-methylcytosine by TET1 promotes active DNA demethylation in the adult brain. Cell.

[16] Auclair G., Weber M. (2012). Mechanisms of DNA methylation and demethylation in mammals. Biochimie.

[17] Dahl C., Grønbæk K., Guldberg P. (2011). Advances in DNA methylation: 5-hydroxymethylcytosine revisited. Clin. Chim. Acta.

[18] Ehrlich M., Lacey M., Karpf A.R. (2013). Epigenetic Alterations in Oncogenesis.

[19] Kohli R.M., Zhang Y. (2013). TET enzymes, TDG and the dynamics of DNA demethylation. Nature.

[20] Jones P.A., Liang G. (2009). Rethinking how DNA methylation patterns are maintained. Nat. Rev. Genet..

[21] Sharma S., de Carvalho D.D., Jeong S., Jones P.A., Liang G. (2011). Nucleosomes containing methylated DNA stabilize DNA methyltransferases 3A/3B and ensure faithful epigenetic inheritance. PLoS Genet..

[22] Jeong S., Liang G., Sharma S., Lin J.C., Choi S.H., Han H., Yoo C.B., Egger G., Yang A.S., Jones P.A. (2009). Selective anchoring of DNA methyltransferases 3A and 3B to nucleosomes containing methylated DNA. Mol. Cell. Biol..

[23] Rose N.R., Klose R.J. (2014). Understanding the relationship between DNA and histone lysine methylation. Biochim. Biophys. Acta.

[24] Nishiyama A., Yamaguchi L., Sharif J., Johmura Y., Kawamura T., Nakanishi K., Shimamura S., Arita K., Kodama T., Ishikawa F. (2013). Uhrf1-dependent H3K23 ubiquitylation couples maintenance DNA methylation and replication. Nature.

[25] Booth M.J., Branco M.R., Ficz G., Oxley D., Krueger F., Reik W., Balasubramanian S. (2012). Quantitative Sequencing of 5-methylcytosine and 5-hydroxymethylcytosine at single-base resolution. Science.

[26] Ponnaluri V.K.C., Maciejewski J.P., Mukherji M. (2013). A mechanistic overview of TET-mediated 5-methylcytosine oxidation. Biochem. Biophys. Res. Commun..

[27] Wu H., D’Alessio A.C., Ito S., Wang Z., Cui K., Zhao K., Sun Y.E., Zhang Y. (2011). Genome-wide analysis of 5-hydroxymethylcytosine distribution reveals its dual function in transcriptional regulation in mouse embryonic stem cells. Genes. Dev..

[28] Stroud H., Feng S., Morey Kinney S., Pradhan S., Jacobsen S.E. (2011). 5-Hydroxymethylcytosine is associated with enhancers and gene bodies in human embryonic stem cells. Genome Biol..

[29] Tan L., Shi Y.G. (2012). Tet family proteins and 5-hydroxymethylcytosine in development and disease. Development.

[30] Okashita N., Kumaki Y., Ebi K., Nishi M., Okamoto Y., Nakayama M., Hashimoto S., Nakamura T., Sugasawa K., Kojima N. (2014). PRDM14 promotes active DNA demethylation through the Ten-eleven translocation (TET)-mediated base excision repair pathway in embryonic stem cells. Development.

[31] Moore S.P., Toomire K.J., Strauss P.R. (2013). DNA modifications repaired by base excision repair are epigenetic. DNA Repair (Amst.).

[32] Solary E., Bernard O.A., Tefferi A., Fuks F., Vainchenker W. (2013). The Ten-Eleven Translocation-2 (TET2) gene in hematopoiesis and hematopoietic diseases. Leukemia.

[33] Blaschke K., Ebata K.T., Karimi M.M., Zepeda-Martínez J.A., Goyal P., Mahapatra S., Tam A., Laird D.J., Hirst M., Rao A. (2013). Vitamin C induces Tet-dependent DNA demethylation and a blastocyst-like state in ES cells. Nature.

[34] Minor E.A., Court B.L., Young J.I., Wang G. (2013). Ascorbate induces ten-eleven translocation (Tet) methylcytosine dioxygenase-mediated generation of 5-hydroxymethylcytosine. J. Biol. Chem..

[35] Liyanage V.R.B., Zachariah R.M., Delcuve G.P., Davie J.R., Rastegar M., Simpson N.M., Stewart V.J. (2012). New Developments in Chromatin Research: An Epigenetic Perspective.

[36] Mellen M., Ayata P., Dewell S., Kriaucionis S., Heintz N. (2012). MeCP2 binds to 5hmC enriched within active genes and accessible chromatin in the nervous system. Cell.

[37] Baron B., Dricu P.A. (2012). Breaking the Silence: The Interplay Between Transcription Factors and DNA Methylation. Methylation—From DNA, RNA and Histones to Diseases and Treatment.

[38] Zhu W.G., Srinivasan K., Dai Z., Duan W., Druhan L.J., Ding H., Yee L., Villalona-Calero M.A., Plass C., Otterson G.A. (2003). Methylation of adjacent CpG sites affects Sp1/Sp3 binding and activity in the p21(Cip1) promoter. Mol. Cell. Biol..

[39] Collings C.K., Waddell P.J., Anderson J.N. (2013). Effects of DNA methylation on nucleosome stability. Nucl. Acids Res..

[40] Lee J.Y., Lee T.H. (2012). Effects of DNA methylation on the structure of nucleosomes. J. Am. Chem. Soc..

[41] Chodavarapu R.K., Feng S., Bernatavichute Y.V., Chen P.Y., Stroud H., Yu Y., Hetzel J.A., Kuo F., Kim J., Cokus S.J. (2010). Relationship between nucleosome positioning and DNA methylation. Nature.

[42] Choy J.S., Wei S., Lee J.Y., Tan S., Chu S., Lee T.H. (2010). DNA methylation increases nucleosome compaction and rigidity. J. Am. Chem. Soc..

[43] Yang X., Noushmehr H., Han H., Andreu-Vieyra C., Liang G., Jones P.A. (2012). Gene reactivation by 5-aza-2ꞌ-deoxycytidine-induced demethylation requires SRCAP-mediated H2A.Z insertion to establish nucleosome depleted regions. PLoS Genet..

[44] Zachariah R.M., Rastegar M. (2012). Linking epigenetics to human disease and Rett syndrome: The emerging novel and challenging concepts in MeCP2 research. Neural Plast..

[45] Hashimoto H., Liu Y., Upadhyay A.K., Chang Y., Howerton S.B., Vertino P.M., Zhang X., Cheng X. (2012). Recognition and potential mechanisms for replication and erasure of cytosine hydroxymethylation. Nucl. Acids Res..

[46] Cartron P.F., Nadaradjane A., Lepape F., Lalier L., Gardie B., Vallette F.M. (2013). Identification of TET1 partners that control its dna-demethylating function. Genes. Cancer.

[47] Zhubi A., Chen Y., Dong E., Cook E.H., Guidotti A., Grayson D.R. (2014). Increased binding of MeCP2 to the GAD1 and RELN promoters may be mediated by an enrichment of 5-hmC in autism spectrum disorder (ASD) cerebellum. Transl. Psychiatry.

[48] Anastasiadou C., Malousi A., Maglaveras N., Kouidou S. (2011). Human epigenome data reveal increased CpG methylation in alternatively spliced sites and putative exonic splicing enhancers. DNA Cell Biol..

[49] Choi J.K. (2010). Contrasting chromatin organization of CpG islands and exons in the human genome. Genome Biol..

[50] Malousi A., Kouidou S. (2012). DNA hypermethylation of alternatively spliced and repeat sequences in humans. Mol. Genet. Genomics.

[51] Maunakea A.K., Chepelev I., Cui K., Zhao K. (2013). Intragenic DNA methylation modulates alternative splicing by recruiting MeCP2 to promote exon recognition. Cell Res..

[52] Long S.W., Ooi J.Y., Yau P.M., Jones P.L. (2011). A brain-derived MeCP2 complex supports a role for MeCP2 in RNA processing. Biosci. Rep..

[53] Young J.I., Hong E.P., Castle J.C., Crespo-Barreto J., Bowman A.B., Rose M.F., Kang D., Richman R., Johnson J.M., Berget S., Zoghbi H.Y. (2005). Regulation of RNA splicing by the methylation-dependent transcriptional repressor methyl-CpG binding protein 2. Proc. Natl. Acad. Sci. USA.

[54] Kornblihtt A.R. (2012). CTCF: From insulators to alternative splicing regulation. Cell Res..

[55] Shukla S., Kavak E., Gregory M., Imashimizu M., Shutinoski B., Kashlev M., Oberdoerffer P., Sandberg R., Oberdoerffer S. (2011). CTCF-promoted RNA polymerase II pausing links DNA methylation to splicing. Nature.

[56] Stadhouders R., Thongjuea S., Andrieu-Soler C., Palstra R.J., Bryne J.C., van den Heuvel A., Stevens M., de Boer E., Kockx C., van der Sloot A. (2012). Dynamic long-range chromatin interactions control Myb proto-oncogene transcription during erythroid development. EMBO J..

[57] Paredes S.H., Melgar M.F., Sethupathy P. (2013). Promoter-proximal CCCTC-factor binding is associated with an increase in the transcriptional pausing index. Bioinformatics.

[58] Kubiura M., Okano M., Kimura H., Kawamura F., Tada M. (2012). Chromosome-wide regulation of euchromatin-specific 5mC to 5hmC conversion in mouse ES cells and female human somatic cells. Chromosom. Res..

[59] Alioui A., Wheldon L.M., Abakir A., Ferjentsik Z., Johnson A.D., Ruzov A. (2012). 5-Carboxylcytosine is localized to euchromatic regions in the nuclei of follicular cells in axolotl ovary. Nucleus.

[60] Shen L., Wu H., Diep D., Yamaguchi S., D’Alessio A.C., Fung H.L., Zhang K., Zhang Y. (2013). Genome-wide analysis reveals TET- and TDG-dependent 5-methylcytosine oxidation dynamics. Cell.

[61] Skene P.J., Illingworth R.S., Webb S., Kerr A.R., James K.D., Turner D.J., Andrews R., Bird A.P. (2010). Neuronal MeCP2 is expressed at near histone-octamer levels and globally alters the chromatin state. Mol. Cell.

[62] Ghosh R.P., Horowitz-Scherer R.A., Nikitina T., Shlyakhtenko L.S., Woodcock C.L. (2010). MeCP2 binds cooperatively to its substrate and competes with histone H1 for chromatin binding sites. Mol. Cell. Biol..

[63] Nikitina T., Ghosh R.P., Horowitz-Scherer R.A., Hansen J.C., Grigoryev S.A., Woodcock C.L. (2007). MeCP2-chromatin interactions include the formation of chromatosome-like structures and are altered in mutations causing Rett syndrome. J. Biol. Chem..

[64] Yasui D.H., Peddada S., Bieda M.C., Vallero R.O., Hogart A., Nagarajan R.P., Thatcher K.N., Farnham P.J., Lasalle J.M. (2007). Integrated epigenomic analyses of neuronal MeCP2 reveal a role for long-range interaction with active genes. Proc. Natl. Acad. Sci. USA.

[65] Nikitina T., Shi X., Ghosh R.P., Horowitz-Scherer R.A., Hansen J.C., Woodcock C.L. (2007). Multiple modes of interaction between the methylated DNA binding protein MeCP2 and chromatin. Mol. Cell. Biol..

[66] Phillips J.E., Corces V.G. (2009). CTCF: Master weaver of the genome. Cell.

[67] Majumder P., Gomez J.A., Chadwick B.P., Boss J.M. (2008). The insulator factor CTCF controls MHC class II gene expression and is required for the formation of long-distance chromatin interactions. J. Exp. Med..

[68] Holwerda S.J., de Laat W. (2013). CTCF: The protein, the binding partners, the binding sites and their chromatin loops. Philos. Trans. R Soc. Lond. B Biol. Sci..

[69] Handoko L., Xu H., Li G., Ngan C.Y., Chew E., Schnapp M., Lee C.W., Ye C., Ping J.L., Mulawadi F. (2011). CTCF-mediated functional chromatin interactome in pluripotent cells. Nat. Genet..

[70] Botta M., Haider S., Leung I.X., Lio P., Mozziconacci J. (2010). Intra- and inter-chromosomal interactions correlate with CTCF binding genome wide. Mol. Syst. Biol..

[71] Gilbert N., Thomson I., Boyle S., Allan J., Ramsahoye B., Bickmore W.A. (2007). DNA methylation affects nuclear organization, histone modifications, and linker histone binding but not chromatin compaction. J. Cell Biol..

[72] Singleton M.K., Gonzales M.L., Leung K.N., Yasui D.H., Schroeder D.I., Dunaway K., LaSalle J.M. (2011). MeCP2 is required for global heterochromatic and nucleolar changes during activity-dependent neuronal maturation. Neurobiol. Dis..

[73] Matarazzo M.R., Boyle S., D’Esposito M., Bickmore W.A. (2007). Chromosome territory reorganization in a human disease with altered DNA methylation. Proc. Natl. Acad. Sci. USA.

[74] Huh I., Zeng J., Park T., Yi S.V. (2013). DNA methylation and transcriptional noise. Epigenetics Chromatin.

[75] Della Ragione F., Filosa S., Scalabri F., D’Esposito M. (2012). MeCP2 as a genome-wide modulator: The renewal of an old story. Front. Genet..

[76] Miura A., Yonebayashi S., Watanabe K., Toyama T., Shimada H., Kakutani T. (2001). Mobilization of transposons by a mutation abolishing full DNA methylation in *Arabidopsis*. Nature.

[77] Slotkin R.K., Martienssen R. (2007). Transposable elements and the epigenetic regulation of the genome. Nat. Rev. Genet..

[78] Leung D., Du T., Wagner U., Xie W., Lee A.Y., Goyal P., Li Y., Szulwach K.E., Jin P., Lorincz M.C., Ren B. (2014). Regulation of DNA methylation turnover at LTR retrotransposons and imprinted loci by the histone methyltransferase Setdb1. Proc. Natl. Acad. Sci..

[79] Lee J., Inoue K., Ono R., Ogonuki N., Kohda T., Kaneko-Ishino T., Ogura A., Ishino F. (2002). Erasing genomic imprinting memory in mouse clone embryos produced from day 11.5 primordial germ cells. Development.

[80] Yamaguchi S., Hong K., Liu R., Inoue A., Shen L., Zhang K., Zhang Y. (2013). Dynamics of 5-methylcytosine and 5-hydroxymethylcytosine during germ cell reprogramming. Cell Res..

[81] Hackett J.A., Sengupta R., Zylicz J.J., Murakami K., Lee C., Down T.A., Surani M.A. (2013). Germline DNA demethylation dynamics and imprint erasure through 5-hydroxymethylcytosine. Science.

[82] Murphy S.K., Huang Z., Hoyo C. (2012). Differentially methylated regions of imprinted genes in prenatal, perinatal and postnatal human tissues. PLoS One.

[83] Coolen M.W., Statham A.L., Qu W., Campbell M.J., Henders A.K., Montgomery G.W., Martin N.G., Clark S.J. (2011). Impact of the genome on the epigenome is manifested in DNA methylation patterns of imprinted regions in monozygotic and dizygotic twins. PLoS One.

[84] Kernohan K.D., Bérubé N.G. (2010). Genetic and epigenetic dysregulation of imprinted genes in the brain. Epigenomics.

[85] Court F., Martin-Trujillo A., Romanelli V., Garin I., Iglesias-Platas I., Salafsky I., Guitart M., Perez de Nanclares G., Lapunzina P., Monk D. (2013). Genome-wide allelic methylation analysis reveals disease-specific susceptibility to multiple methylation defects in imprinting syndromes. Hum. Mutat..

[86] Soubry A., Murphy S.K., Wang F., Huang Z., Vidal A.C., Fuemmeler B.F., Kurtzberg J., Murtha A., Jirtle R.L., Schildkraut J.M. (2013). Newborns of obese parents have altered DNA methylation patterns at imprinted genes. Int. J. Obes..

[87] LaSalle J.M. (2007). The Odyssey of MeCP2 and parental imprinting. Epigenetics.

[88] Flashner B.M., Russo M.E., Boileau J.E., Leong D.W., Gallicano G.I. (2013). Epigenetic factors and autism spectrum disorders. NeuroMol. Med..

[89] Lewis A., Murrell A. (2004). Genomic imprinting: CTCF protects the boundaries. Curr. Biol..

[90] Prickett A.R., Barkas N., McCole R.B., Hughes S., Amante S.M., Schulz R., Oakey R.J. (2013). Genome-wide and parental allele-specific analysis of CTCF and cohesin DNA binding in mouse brain reveals a tissue-specific binding pattern and an association with imprinted differentially methylated regions. Genome Res..

[91] Zuo X., Sheng J., Lau H.-T., McDonald C.M., Andrade M., Cullen D.E., Bell F.T., Iacovino M., Kyba M., Xu G. (2012). Zinc finger protein ZFP57 requires its co-factor to recruit DNA methyltransferases and maintains DNA methylation imprint in embryonic stem cells via its transcriptional repression domain. J. Biol. Chem..

[92] Quenneville S., Verde G., Corsinotti A., Kapopoulou A., Jakobsson J., Offner S., Baglivo I., Pedone P.V., Grimaldi G., Riccio A. (2011). In embryonic stem cells, ZFP57/KAP1 recognize a methylated hexanucleotide to affect chromatin and DNA methylation of imprinting control regions. Mol. Cell.

[93] Pontier D.B., Gribnau J. (2011). Xist regulation and function explored. Hum. Genet..

[94] Marks H., Chow J.C., Denissov S., Françoijs K.-J., Brockdorff N., Heard E., Stunnenberg H.G. (2009). High-resolution analysis of epigenetic changes associated with X inactivation. Genome Res..

[95] Ahn J.Y., Lee J.T. (2008). X chromosome: X inactivation. Nat. Educ..

[96] Escamilla-Del-Arenal M., da Rocha S.T., Heard E. (2011). Evolutionary diversity and developmental regulation of X-chromosome inactivation. Hum. Genet..

[97] Sharp A.J., Stathaki E., Migliavacca E., Brahmachary M., Montgomery S.B., Dupre Y., Antonarakis S.E. (2011). DNA methylation profiles of human active and inactive X chromosomes. Genome Res..

[98] Hellman A., Chess A. (2007). Gene body-specific methylation on the active X chromosome. Science.

[99] Migeon B.R., Chowdhury A.K., Dunston J.A., McIntosh I. (2001). Identification of TSIX, encoding an RNA antisense to human XIST, reveals differences from its murine counterpart: Implications for X inactivation. Am. J. Hum. Genet..

[100] Neri F., Krepelova A., Incarnato D., Maldotti M., Parlato C., Galvagni F., Matarese F., Stunnenberg H.G., Oliviero S. (2013). Dnmt3L antagonizes DNA methylation at bivalent promoters and favors DNA methylation at gene bodies in ESCs. Cell.

[101] Brinkman A.B., Gu H., Bartels S.J., Zhang Y., Matarese F., Simmer F., Marks H., Bock C., Gnirke A., Meissner A. (2012). Sequential ChIP-bisulfite sequencing enables direct genome-scale investigation of chromatin and DNA methylation cross-talk. Genome Res..

[102] Nesterova T.B., Popova B.C., Cobb B.S., Norton S., Senner C.E., Tang Y.A., Spruce T., Rodriguez T.A., Sado T., Merkenschlager M. (2008). Dicer regulates Xist promoter methylation in ES cells indirectly through transcriptional control of Dnmt3a. Epigenetics Chromatin.

[103] Navarro P., Pichard S., Ciaudo C., Avner P., Rougeulle C. (2005). Tsix transcription across the Xist gene alters chromatin conformation without affecting Xist transcription: Implications for X-chromosome inactivation. Genes Dev..

[104] Chao W., Huynh K.D., Spencer R.J., Davidow L.S., Lee J.T. (2002). CTCF, a candidate trans-acting factor for X-inactivation choice. Science.

[105] Zhou Q., Teng F. (2013). Epigenetic re-programming during mammalian preimplantation embryogenesis and pgc development. J. IVF Reprod Med. Genet..

[106] Lan J., Hua S., He X., Zhang Y. (2010). DNA methyltransferases and methyl-binding proteins of mammals. Acta Biochim. Biophys. Sin..

[107] Saito M., Ishikawa F. (2002). The mCpG-binding domain of human MBD3 does not bind to mCpG but interacts with NuRD/Mi2 components HDAC1 and MTA2. J. Biol. Chem..

[108] Prokhortchouk A., Hendrich B., Jorgensen H., Ruzov A., Wilm M., Georgiev G., Bird A., Prokhortchouk E. (2001). The p120 catenin partner Kaiso is a DNA methylation-dependent transcriptional repressor. Genes Dev..

[109] Liyanage V.R., Rastegar M. (2014). Rett Syndrome and MeCP2. Neuromol. Med..

[110] Fraga M.F., Ballestar E., Montoya G., Taysavang P., Wade P.A., Esteller M. (2003). The affinity of different MBD proteins for a specific methylated locus depends on their intrinsic binding properties. Nucl. Acids Res..

[111] Klose R.J., Sarraf S.A., Schmiedeberg L., McDermott S.M., Stancheva I., Bird A.P. (2005). DNA binding selectivity of MeCP2 due to a requirement for A/T sequences adjacent to methyl-CpG. Mol. Cell.

[112] Clouaire T., de Las Heras J.I., Merusi C., Stancheva I. (2010). Recruitment of MBD1 to target genes requires sequence-specific interaction of the MBD domain with methylated DNA. Nucl. Acids Res..

[113] Scarsdale J.N., Webb H.D., Ginder G.D., Williams D.C. (2011). Solution structure and dynamic analysis of chicken MBD2 methyl binding domain bound to a target-methylated DNA sequence. Nucl. Acids Res..

[114] Spruijt C.G., Gnerlich F., Smits A.H., Pfaffeneder T., Jansen P.W., Bauer C., Munzel M., Wagner M., Muller M., Khan F. (2013). Dynamic readers for 5-(hydroxy)methylcytosine and its oxidized derivatives. Cell.

[115] Iurlaro M., Ficz G., Oxley D., Raiber E.A., Bachman M., Booth M.J., Andrews S., Balasubramanian S., Reik W. (2013). A screen for hydroxymethylcytosine and formylcytosine binding proteins suggests functions in transcription and chromatin regulation. Genome Biol.

[116] Yildirim O., Li R., Hung J.H., Chen P.B., Dong X., Ee L.S., Weng Z., Rando O.J., Fazzio T.G. (2011). Mbd3/NURD complex regulates expression of 5-hydroxymethylcytosine marked genes in embryonic stem cells. Cell.

[117] Baubec T., Ivanek R., Lienert F., Schubeler D. (2013). Methylation-dependent and -independent genomic targeting principles of the MBD protein family. Cell.

[118] Hansen J.C., Ghosh R.P., Woodcock C.L. (2010). Binding of the Rett syndrome protein, MeCP2, to methylated and unmethylated DNA and chromatin. IUBMB Life.

[119] Ghosh R.P., Nikitina T., Horowitz-Scherer R.A., Gierasch L.M., Uversky V.N., Hite K., Hansen J.C., Woodcock C.L. (2010). Unique physical properties and interactions of the domains of methylated DNA binding protein 2. Biochemistry.

[120] Jorgensen H.F., Ben-Porath I., Bird A.P. (2004). Mbd1 is recruited to both methylated and nonmethylated CpGs via distinct DNA binding domains. Mol. Cell. Biol..

[121] Cramer J.M., Scarsdale J.N., Walavalkar N.M., Buchwald W.A., Ginder G.D., Williams D.C. (2014). Probing the dynamic distribution of bound states for methylcytosine-binding domains on DNA. J. Biol. Chem..

[122] Parry L., Clarke A.R. (2011). The Roles of the Methyl-CpG Binding Proteins in Cancer. Genes Cancer.

[123] Kimura H., Shiota K. (2003). Methyl-CpG-binding protein, MeCP2, is a target molecule for maintenance DNA methyltransferase, Dnmt1. J. Biol. Chem..

[124] Heard E., Martienssen R.A. (2014). Transgenerational epigenetic inheritance: Myths and mechanisms. Cell.

[125] Dayeh T.A., Olsson A.H., Volkov P., Almgren P., Ronn T., Ling C. (2013). Identification of CpG-SNPs associated with type 2 diabetes and differential DNA methylation in human pancreatic islets. Diabetologia.

[126] Taqi M.M., Bazov I., Watanabe H., Sheedy D., Harper C., Alkass K., Druid H., Wentzel P., Nyberg F., Yakovleva T., Bakalkin G. (2011). Prodynorphin CpG-SNPs associated with alcohol dependence: Elevated methylation in the brain of human alcoholics. Addict. Biol..

[127] Chik F., Szyf M., Rabbani S.A. (2011). Role of epigenetics in cancer initiation and progression. Adv. Exp. Med. Biol..

[128] Yaqinuddin A., Abbas F., Naqvi S.Z., Bashir M.U., Qazi R., Qureshi S.A. (2008). Silencing of MBD1 and MeCP2 in prostate-cancer-derived PC3 cells produces differential gene expression profiles and cellular phenotypes. Biosci. Rep..

[129] Fournier A., Sasai N., Nakao M., Defossez P.A. (2012). The role of methyl-binding proteins in chromatin organization and epigenome maintenance. Br. Funct. Genomics..

[130] Wang Z., Zhang J., Zhang Y., Srivenugopal K.S., Lim S.H. (2006). SPAN-XB core promoter sequence is regulated in myeloma cells by specific CpG dinucleotides associated with the MeCP2 protein. Int. J. Cancer.

[131] Pontes T.B., Chen E.S., Gigek C.O., Calcagno D.Q., Wisnieski F., Leal M.F., Demachki S., Assumpcao P.P., Artigiani R., Lourenco L.G. (2013). Reduced mRNA expression levels of MBD2 and MBD3 in gastric carcinogenesis. Tumour Biol..

[132] Sapkota Y., Robson P., Lai R., Cass C.E., Mackey J.R., Damaraju S. (2012). A two-stage association study identifies methyl-CpG-binding domain protein 2 gene polymorphisms as candidates for breast cancer susceptibility. Eur. J. Hum. Genet..

[133] Stefanska B., Suderman M., Machnes Z., Bhattacharyya B., Hallett M., Szyf M. (2013). Transcription onset of genes critical in liver carcinogenesis is epigenetically regulated by methylated DNA-binding protein MBD2. Carcinogenesis.

[134] Zhao S., Choi M., Overton J.D., Bellone S., Roque D.M., Cocco E., Guzzo F., English D.P., Varughese J., Gasparrini S. (2013). Landscape of somatic single-nucleotide and copy-number mutations in uterine serous carcinoma. Proc. Natl. Acad. Sci. USA.

[135] Hendrich B., Guy J., Ramsahoye B., Wilson V.A., Bird A. (2001). Closely related proteins MBD2 and MBD3 play distinctive but interacting roles in mouse development. Genes Dev..

[136] Kaji K., Caballero I.M., MacLeod R., Nichols J., Wilson V.A., Hendrich B. (2006). The NuRD component Mbd3 is required for pluripotency of embryonic stem cells. Nat. Cell Biol..

[137] Xiong X.D., Luo X.P., Liu X., Jing X., Zeng L.Q., Lei M., Hong X.S., Chen Y. (2012). The MBD4 Glu346Lys polymorphism is associated with the risk of cervical cancer in a Chinese population. Int. J. Gynecol. Cancer.

[138] Jones J., Wang H., Karanam B., Theodore S., Dean-Colomb W., Welch D.R., Grizzle W., Yates C. (2014). Nuclear localization of Kaiso promotes the poorly differentiated phenotype and EMT in infiltrating ductal carcinomas. Clin. Exp. Metastasis.

[139] Vermeulen J.F., van de Ven R.A., Ercan C., van der Groep P., van der Wall E., Bult P., Christgen M., Lehmann U., Daniel J., van Diest P.J. (2012). Nuclear Kaiso expression is associated with high grade and triple-negative invasive breast cancer. PLoS One.

[140] Dai S.D., Wang Y., Jiang G.Y., Zhang P.X., Dong X.J., Wei Q., Xu H.T., Li Q.C., Zhao C., Wang E.H. (2010). Kaiso is expressed in lung cancer: Its expression and localization is affected by p120ctn. Lung Cancer.

[141] Ling C., Groop L. (2009). Epigenetics: A molecular link between environmental factors and type 2 diabetes. Diabetes.

[142] Shaikh M.K., Devrajani B.R., Shaikh A., Shah S.Z.A., Shaikh S., Singh D. (2012). Plasma homocysteine level in patients with diabetes mellitus. World Appl. Sci. J..

[143] Zheng M., Zhang M., Yang J., Zhao S., Qin S., Chen H., Gao Y., Huang G. (2014). Relationship between blood levels of methyl donor and folate and mild cognitive impairment in Chinese patients with type 2 diabetes: A case-control study. J. Clin. Biochem. Nutr..

[144] Al-Maskari M.Y., Waly M.I., Ali A., Al-Shuaibi Y.S., Ouhtit A. (2012). Folate and vitamin B12 deficiency and hyperhomocysteinemia promote oxidative stress in adult type 2 diabetes. Nutrition.

[145] Dominguez R.O., Marschoff E.R., Guareschi E.M., Famulari A.L., Pagano M.A., Serra J.A. (2005). Homocysteine, vitamin B12 and folate in Alzheimer’s and vascular dementias: The paradoxical effect of the superimposed type II diabetes mellitus condition. Clin. Chim. Acta.

[146] Belot M.-P., Fradin D., Mai N., Le Fur S., Zélénika D., Kerr-Conte J., Pattou F., Lucas B., Bougnères P. (2013). CpG methylation changes within the IL2RA promoter in type 1 diabetes of childhood onset. PLoS One.

[147] Ling C., del Guerra S., Lupi R., Ronn T., Granhall C., Luthman H., Masiello P., Marchetti P., Groop L., del Prato S. (2008). Epigenetic regulation of PPARGC1A in human type 2 diabetic islets and effect on insulin secretion. Diabetologia.

[148] Hall E., Dayeh T., Kirkpatrick C.L., Wollheim C.B., Dekker Nitert M., Ling C. (2013). DNA methylation of the glucagon-like peptide 1 receptor (GLP1R) in human pancreatic islets. BMC Med. Genet..

[149] Yang B.T., Dayeh T.A., Volkov P.A., Kirkpatrick C.L., Malmgren S., Jing X., Renstrom E., Wollheim C.B., Nitert M.D., Ling C. (2012). Increased DNA methylation and decreased expression of PDX-1 in pancreatic islets from patients with type 2 diabetes. Mol. Endocrinol..

[150] Zhang H., Cai X., Yi B., Huang J., Wang J., Sun J. (2014). Correlation of CTGF gene promoter methylation with CTGF expression in type 2 diabetes mellitus with or without nephropathy. Mol. Med. Rep..

[151] Kuroda A., Rauch T.A., Todorov I., Ku H.T., Al-Abdullah I.H., Kandeel F., Mullen Y., Pfeifer G.P., Ferreri K. (2009). Insulin gene expression is regulated by DNA methylation. PLoS One.

[152] Pitcher M.R., Ward C.S., Arvide E.M., Chapleau C.A., Pozzo-Miller L., Hoeflich A., Sivaramakrishnan M., Saenger S., Metzger F., Neul J.L. (2013). Insulinotropic treatments exacerbate metabolic syndrome in mice lacking MeCP2 function. Hum. Mol. Genet..

[153] Akin L., Adal E., Akin M.A., Kurtoglu S. (2012). A case of diabetes mellitus associated with Rett syndrome. J. Pediatr. Endocrinol. Metab..

[154] Rekik N.M., Kamoun M., Mnif F., Charfi N., Mnif M.F., Abid M. (2010). Type 1 diabetes mellitus and Rett syndrome: Is there a link?. J. Endocrinol. Invest..

[155] Kurtoglu S., Atabek M.E., Kumandas S., Keskin M. (2005). Diabetes mellitus type 1: Association with Rett syndrome. Pediatr. Int..

[156] Butler M.G. (2009). Genomic imprinting disorders in humans: A mini-review. J. Assist. Reprod. Genet..

[157] Sharp A.J., Migliavacca E., Dupre Y., Stathaki E., Sailani M.R., Baumer A., Schinzel A., Mackay D.J., Robinson D.O., Cobellis G. (2010). Methylation profiling in individuals with uniparental disomy identifies novel differentially methylated regions on chromosome 15. Genome Res..

[158] Dias R.P., Bogdarina I., Cazier J.B., Buchanan C., Donaldson M.C., Johnston L.B., Hokken-Koelega A.C., Clark A.J. (2012). Multiple segmental uniparental disomy associated with abnormal DNA methylation of imprinted Loci in silver-russell syndrome. J. Clin. Endocrinol. Metab..

[159] Han K., Gennarino V.A., Lee Y., Pang K., Hashimoto-Torii K., Choufani S., Raju C.S., Oldham M.C., Weksberg R., Rakic P., Liu Z., Zoghbi H.Y. (2013). Human-specific regulation of MeCP2 levels in fetal brains by microRNA miR-483-5p. Genes Dev..

[160] Drewell R.A., Goddard C.J., Thomas J.O., Surani M.A. (2002). Methylation-dependent silencing at the H19 imprinting control region by MeCP2. Nucl. Acids Res..

[161] Fuks F., Hurd P.J., Wolf D., Nan X., Bird A.P., Kouzarides T. (2003). The methyl-CpG-binding protein MeCP2 links DNA methylation to histone methylation. J. Biol. Chem..

[162] Makedonski K., Abuhatzira L., Kaufman Y., Razin A., Shemer R. (2005). MeCP2 deficiency in Rett syndrome causes epigenetic aberrations at the PWS/AS imprinting center that affects UBE3A expression. Hum. Mol. Genet..

[163] Samaco R.C., Hogart A., LaSalle J.M. (2005). Epigenetic overlap in autism-spectrum neurodevelopmental disorders: MECP2 deficiency causes reduced expression of UBE3A and GABRB3. Hum. Mol. Genet..

[164] Horike S., Cai S., Miyano M., Cheng J.F., Kohwi-Shigematsu T. (2005). Loss of silent-chromatin looping and impaired imprinting of DLX5 in Rett syndrome. Nat. Genet..

[165] Shukla S.D., Velazquez J., French S.W., Lu S.C., Ticku M.K., Zakhari S. (2008). Emerging role of epigenetics in the actions of alcohol. Alcohol. Clin. Exp. Res..

[166] Wang T., Nandakumar V., Jiang X.X., Jones L., Yang A.G., Huang X.F., Chen S.Y. (2013). The control of hematopoietic stem cell maintenance, self-renewal, and differentiation by Mysm1-mediated epigenetic regulation. Blood.

[167] Beerman I., Bock C., Garrison B.S., Smith Z.D., Gu H., Meissner A., Rossi D.J. (2013). Proliferation-dependent alterations of the DNA methylation landscape underlie hematopoietic stem cell aging. Cell Stem Cell.

[168] Hogart A., Lichtenberg J., Ajay S.S., Anderson S., Margulies E.H., Bodine D.M. (2012). Genome-wide DNA methylation profiles in hematopoietic stem and progenitor cells reveal overrepresentation of ETS transcription factor binding sites. Genome Res..

[169] Suarez-Alvarez B., Rodriguez R.M., Fraga M.F., Lopez-Larrea C. (2012). DNA methylation: A promising landscape for immune system-related diseases. Trends Genet..

[170] Friez M.J., Jones J.R., Clarkson K., Lubs H., Abuelo D., Bier J.A., Pai S., Simensen R., Williams C., Giampietro P.F., Schwartz C.E., Stevenson R.E. (2006). Recurrent infections, hypotonia, and mental retardation caused by duplication of MECP2 and adjacent region in Xq28. Pediatrics.

[171] Yang T., Ramocki M.B., Neul J.L., Lu W., Roberts L., Knight J., Ward C.S., Zoghbi H.Y., Kheradmand F., Corry D.B. (2012). Overexpression of methyl-CpG binding protein 2 impairs T(H)1 responses. Sci. Transl. Med..

[172] Sawalha A.H., Webb R., Han S., Kelly J.A., Kaufman K.M., Kimberly R.P., Alarcon-Riquelme M.E., James J.A., Vyse T.J., Gilkeson G.S. (2008). Common variants within MECP2 confer risk of systemic lupus erythematosus. PLoS One.

[173] Sawalha A.H. (2013). Overexpression of methyl-CpG-binding protein 2 and autoimmunity: Evidence from MECP2 duplication syndrome, lupus, MECP2 transgenic and Mecp2 deficient mice. Lupus.

[174] Cobb B.L., Fei Y., Jonsson R., Bolstad A.I., Brun J.G., Rischmueller M., Lester S.E., Witte T., Illei G., Brennan M. (2010). Genetic association between methyl-CpG binding protein 2 (MECP2) and primary Sjogren’s syndrome. Ann. Rheum. Dis..

[175] Szyf M. (2010). Epigenetic therapeutics in autoimmune disease. Clin. Rev. Allergy Immunol..

[176] Balada E., Ordi-Ros J., Vilardell-Tarres M. (2007). DNA methylation and systemic lupus erythematosus. Ann. N. Y. Acad. Sci..

[177] Balada E., Ordi-Ros J., Serrano-Acedo S., Martinez-Lostao L., Vilardell-Tarres M. (2007). Transcript overexpression of the MBD2 and MBD4 genes in CD4+ T cells from systemic lupus erythematosus patients. J. Leukoc. Biol..

[178] Udali S., Guarini P., Moruzzi S., Choi S.W., Friso S. (2013). Cardiovascular epigenetics: From DNA methylation to microRNAs. Mol. Aspects Med..

[179] Handy D.E., Castro R., Loscalzo J. (2011). Epigenetic modifications: Basic mechanisms and role in cardiovascular disease. Circulation.

[180] Baccarelli A., Wright R., Bollati V., Litonjua A., Zanobetti A., Tarantini L., Sparrow D., Vokonas P., Schwartz J. (2010). Ischemic heart disease and stroke in relation to blood DNA methylation. Epidemiology.

[181] Endres M., Meisel A., Biniszkiewicz D., Namura S., Prass K., Ruscher K., Lipski A., Jaenisch R., Moskowitz M.A., Dirnagl U. (2000). DNA methyltransferase contributes to delayed ischemic brain injury. J. Neurosci..

[182] Endres M., Fan G., Meisel A., Dirnagl U., Jaenisch R. (2001). Effects of cerebral ischemia in mice lacking DNA methyltransferase 1 in post-mitotic neurons. Neuroreport.

[183] Lin R.-T., Hsi E., Lin H.-F., Liao Y.-C., Wang Y.-S., Juo S.-H. (2014). LINE-1 methylation is associated with an increased risk of ischemic stroke in men. Curr. Neurovasc. Res..

[184] Schweizer S., Meisel A., Märschenz S. (2013). Epigenetic mechanisms in cerebral ischemia. J. Cereb. Blood Flow Metab..

[185] Lusardi T.A., Farr C.D., Faulkner C.L., Pignataro G., Yang T., Lan J., Simon R.P., Saugstad J.A. (2010). Ischemic preconditioning regulates expression of microRNAs and a predicted target, MeCP2, in mouse cortex. J. Cereb. Blood Flow Metab..

[186] Gan H., Wen L., Liao S., Lin X., Ma T., Liu J., Song C.X., Wang M., He C., Han C. (2013). Dynamics of 5-hydroxymethylcytosine during mouse spermatogenesis. Nat. Commun..

[187] Klenova E.M., Nicolas R.H., Paterson H.F., Carne A.F., Heath C.M., Goodwin G.H., Neiman P.E., Lobanenkov V.V. (1993). CTCF, a conserved nuclear factor required for optimal transcriptional activity of the chicken c-myc gene, is an 11-Zn-finger protein differentially expressed in multiple forms. Mol. Cell. Biol..

[188] Szabo P.E., Tang S.H., Silva F.J., Tsark W.M., Mann J.R. (2004). Role of CTCF binding sites in the Igf2/H19 imprinting control region. Mol. Cell. Biol..

[189] Zlatanova J., Caiafa P. (2009). CTCF and its protein partners: Divide and rule?. J. Cell Sci..

[190] Gause M., Schaaf C.A., Dorsett D. (2008). Cohesin and CTCF: Cooperating to control chromosome conformation?. Bioessays.

[191] Chernukhin I., Shamsuddin S., Kang S.Y., Bergstrom R., Kwon Y.W., Yu W., Whitehead J., Mukhopadhyay R., Docquier F., Farrar D. (2007). CTCF interacts with and recruits the largest subunit of RNA polymerase II to CTCF target sites genome-wide. Mol. Cell. Biol..

[192] Donohoe M.E., Zhang L.F., Xu N., Shi Y., Lee J.T. (2007). Identification of a Ctcf cofactor, YY1, for the X chromosome binary switch. Mol. Cell.

[193] Aulmann S., Blaker H., Penzel R., Rieker R.J., Otto H.F., Sinn H.P. (2003). CTCF gene mutations in invasive ductal breast cancer. Breast Cancer Res. Treat..

[194] Docquier F., Farrar D., D’Arcy V., Chernukhin I., Robinson A.F., Loukinov D., Vatolin S., Pack S., Mackay A., Harris R.A. (2005). Heightened expression of CTCF in breast cancer cells is associated with resistance to apoptosis. Cancer Res..

[195] Paradowska A., Fenic I., Konrad L., Sturm K., Wagenlehner F., Weidner W., Steger K. (2009). Aberrant epigenetic modifications in the CTCF binding domain of the IGF2/H19 gene in prostate cancer compared with benign prostate hyperplasia. Int. J. Oncol..

[196] Fiorentino F.P., Macaluso M., Miranda F., Montanari M., Russo A., Bagella L., Giordano A. (2011). CTCF and BORIS regulate Rb2/p130 gene transcription: A novel mechanism and a new paradigm for understanding the biology of lung cancer. Mol. Cancer Res..

[197] Nakagawa H., Chadwick R.B., Peltomaki P., Plass C., Nakamura Y., de La Chapelle A. (2001). Loss of imprinting of the insulin-like growth factor II gene occurs by biallelic methylation in a core region of H19-associated CTCF-binding sites in colorectal cancer. Proc. Natl. Acad. Sci. USA.

[198] Weksberg R., Smith A.C., Squire J., Sadowski P. (2003). Beckwith-Wiedemann syndrome demonstrates a role for epigenetic control of normal development. Hum. Mol. Genet..

[199] Nagarajan R.P., Patzel K.A., Martin M., Yasui D.H., Swanberg S.E., Hertz-Picciotto I., Hansen R.L., van de Water J., Pessah I.N., Jiang R., Robinson W.P., LaSalle J.M. (2008). MECP2 promoter methylation and X chromosome inactivation in autism. Autism Res..

[200] Stiles J., Jernigan T.L. (2010). The basics of brain development. Neuropsychol. Rev..

[201] Sadler T.W. (2005). Embryology of neural tube development. Am. J. Med. Genet. C. Semin. Med. Genet..

[202] O’Rahilly R.R., Muller F. (1999). The Embryonic Human Brain: An Atlas of Developmental Stages.

[203] Morgan H.D., Santos F., Green K., Dean W., Reik W. (2005). Epigenetic reprogramming in mammals. Hum. Mol. Genet..

[204] Seisenberger S., Peat J.R., Hore T.A., Santos F., Dean W., Reik W. (2013). Reprogramming DNA methylation in the mammalian life cycle: Building and breaking epigenetic barriers. Philasophical Trans. R. Soc. Biol. Sci..

[205] Zhou F.C. (2012). DNA methylation program during development. Front. Biol. (Beijing).

[206] Chen Y., Damayanti N.P., Irudayaraj J., Dunn K., Zhou F.C. (2014). Diversity of two forms of DNA methylation in the brain. Front. Genet..

[207] Chen Y., Ozturk N.C., Zhou F.C. (2013). DNA methylation program in developing hippocampus and its alteration by alcohol. PLoS One.

[208] Lister R., Mukamel E.A., Nery J.R., Urich M., Puddifoot C.A., Johnson N.D., Lucero J., Huang Y., Dwork A.J., Schultz M.D. (2013). Global epigenomic reconfiguration during mammalian brain development. Science.

[209] Guo J.U., Su Y., Shin J.H., Shin J., Li H., Xie B., Zhong C., Hu S., Le T., Fan G. (2014). Distribution, recognition and regulation of non-CpG methylation in the adult mammalian brain. Nat. Neurosci..

[210] LaSalle J.M. (2011). A genomic point-of-view on environmental factors influencing the human brain methylome. Epigenetics.

[211] Perera F., Herbstman J. (2011). Prenatal environmental exposures, epigenetics, and disease. Reprod. Toxicol..

[212] Israel Y., Rivera-Meza M., Karahanian E., Quintanilla M.E., Tampier L., Morales P., Herrera-Marschitz M. (2013). Gene specific modifications unravel ethanol and acetaldehyde actions. Front. Behav. Neurosci..

[213] Behnke M., Smith V.C. (2013). Prenatal substance abuse: Short- and long-term effects on the exposed fetus. Pediatrics.

[214] Tian W., Zhao M., Li M., Song T., Zhang M., Quan L., Li S., Sun Z.S. (2012). Reversal of cocaine-conditioned place preference through methyl supplementation in mice: Altering global DNA methylation in the prefrontal cortex. PLoS One.

[215] Satta R., Maloku E., Zhubi A., Pibiri F., Hajos M., Costa E., Guidotti A. (2008). Nicotine decreases DNA methyltransferase 1 expression and glutamic acid decarboxylase 67 promoter methylation in GABAergic interneurons. PNAS.

[216] Liu Y., Balaraman Y., Wang G., Nephew K.P., Zhou F.C. (2009). Alcohol exposure alters DNA methylation profiles in mouse embryos at early neurulation. Epigenetics.

[217] Zhou F.C., Balaraman Y., Teng M., Liu Y., Singh R.P., Nephew K.P. (2011). Alcohol alters DNA methylation patterns and inhibits neural stem cell differentiation. Alcohol. Clin. Exp. Res..

[218] Varela-Rey M., Woodhoo A., Martinez-Chantar M.-L., Mato J.M., Lu S.C. (2011). Alcohol, DNA methylation, and cancer. Alcohol Res. Curr. Rev..

[219] Kruman I., Fowler A.-K. (2014). One-carbon metabolism and risk factors for its disturbance. J. Neurochem..

[220] Laufer B.I., Mantha K., Kleiber M.L., Diehl E.J., Addison S.M.F., Singh S.M. (2013). Long-lasting alterations to DNA methylation and ncRNAs could underlie the effects of fetal alcohol exposure in mice. Dis. Model. Mech..

[221] Bielawski D.M., Zaher F.M., Svinarich D.M., Abel E.L. (2002). Paternal alcohol exposure affects sperm cytosine methyltransferase messenger RNA levels. Alcohol. Clin. Exp. Res..

[222] Ouko L.A., Shantikumar K., Knezovich J., Haycock P., Schnugh D.J., Ramsay M. (2009). Effect of alcohol consumption on CpG methylation in the differentially methylated regions of H19 and IG-DMR in male gametes: Implications for fetal alcohol spectrum disorders. Alcohol. Clin. Exp. Res..

[223] Stouder C., Somm E., Paoloni-Giacobino A. (2011). Prenatal exposure to ethanol: A specific effect on the H19 gene in sperm. Reprod. Toxicol..

[224] Knezovich J.G., Ramsay M. (2012). The effect of preconception paternal alcohol exposure on epigenetic remodeling of the h19 and rasgrf1 imprinting control regions in mouse offspring. Front. Genet..

[225] Maccani J.Z., Koestler D.C., Houseman E.A., Marsit C.J., Kelsey K.T. (2013). Placental DNA methylation alterations associated with maternal tobacco smoking at the RUNX3 gene are also associated with gestational age. Epigenomics.

[226] Joubert B.R., Håberg S.E., Nilsen R.M., Wang X., Vollset S.E., Murphy S.K., Nystad W., Bell D.A., Peddada S.D., London S.J. (2012). 450K epigenome-wide scan identifies differential DNA methylation in newborns related to maternal smoking during pregnancy. Environ. Health Perspect..

[227] Li Y., Xiao D., Dasgupta C., Xiong F., Tong W., Yang S., Zhang L. (2012). Perinatal nicotine exposure increases vulnerability of hypoxic-ischemic brain injury in neonatal rats: Role of angiotensin II receptors. Stroke.

[228] Breton C.V., Byun H.-M., Wenten M., Pan F., Yang A., Gilliland F.D. (2009). Prenatal tobacco smoke exposure affects global and gene-specific DNA methylation. Am. J. Respir. Crit. Care Med..

[229] Toledo-Rodriguez M., Lotfipour S., Leonard G., Perron M., Richer L., Veillette S., Pausova Z., Paus T. (2010). Maternal smoking during pregnancy is associated with epigenetic modifications of the brain-derived neurotrophic factor-6 exon in adolescent offspring. Am. J. Med. Genet. B. Neuropsychiatr. Genet..

[230] Chung W.C., Auger A.P. (2013). Gender differences in neurodevelopment and epigenetics. Pflugers Arch..

[231] Morrison K.E., Rodgers A.B., Morgan C.P., Bale T.L. (2014). Epigenetic mechanisms in pubertal brain maturation. Neuroscience.

[232] Pagani L.S. (2014). Environmental tobacco smoke exposure and brain development: The case of attention deficit/hyperactivity disorder. Neurosci. Biobehav. Rev..

[233] Novikova S.I., He F., Bai J., Cutrufello N.J., Lidow M.S., Undieh A.S. (2008). Maternal cocaine administration in mice alters DNA methylation and gene expression in hippocampal neurons of neonatal and prepubertal offspring. PLoS One.

[234] Anier K., Malinovskaja K., Aonurm-Helm A., Zharkovsky A., Kalda A. (2010). DNA methylation regulates cocaine-induced behavioral sensitization in mice. Neuropsychopharmacology.

[235] Song L., James S.R., Kazim L., Karpf A.R. (2005). Specific method for the determination of genomic DNA methylation by liquid chromatography-electrospray ionization tandem mass spectrometry. Anal. Chem..

[236] Itzhak Y., Ergui I., Young J.I. (2014). Long-term parental methamphetamine exposure of mice influences behavior and hippocampal DNA methylation of the offspring. Mol. Psychiatry.

[237] Fragou D., Zanos P., Kouidou S., Njau S., Kitchen I., Bailey A., Kovatsi L. (2013). Effect of chronic heroin and cocaine administration on global DNA methylation in brain and liver. Toxicol. Lett..

[238] Clark C., Palta P., Joyce C.J., Scott C., Grundberg E., Deloukas P., Palotie A., Coffey A.J. (2012). A comparison of the whole genome approach of MeDIP-seq to the targeted approach of the Infinium HumanMethylation450 BeadChip^®^ for methylome profiling. PLoS One.

[239] Olynik B.M., Rastegar M. (2012). The genetic and epigenetic journey of embryonic stem cells into mature neural cells. Front. Genet..

[240] Ezeonwuka C., Rastegar M. (2014). MeCP2-related diseases and animal models. Diseases.

[241] Shahbazian M.D., Antalffy B., Armstrong D.L., Zoghbi H.Y. (2002). Insight into Rett syndrome: MeCP2 levels display tissue- and cell-specific differences and correlate with neuronal maturation. Hum. Mol. Genet..

[242] Olson C.O., Zachariah R.M., Ezeonwuka C.D., Liyanage V.R., Rastegar M. (2014). Brain region-specific expression of MeCP2 isoforms correlates with DNA methylation within Mecp2 regulatory elements. PLoS One.

[243] Liyanage V.R., Zachariah R.M., Rastegar M. (2013). Decitabine alters the expression of Mecp2 isoforms via dynamic DNA methylation at the Mecp2 regulatory elements in neural stem cells. Mol. Autism.

[244] Rastegar M., Hotta A., Pasceri P., Makarem M., Cheung A.Y., Elliott S., Park K.J., Adachi M., Jones F.S., Clarke I.D. (2009). MECP2 isoform-specific vectors with regulated expression for Rett syndrome gene therapy. PLoS One.

[245] Renieri A., Meloni I., Longo I., Ariani F., Mari F., Pescucci C., Cambi F. (2003). Rett syndrome: The complex nature of a monogenic disease. J. Mol. Med..

[246] Amir R.E., van den Veyver I.B., Wan M., Tran C.Q., Francke U., Zoghbi H.Y. (1999). Rett syndrome is caused by mutations in X-linked MECP2, encoding methyl-CpG-binding protein 2. Nat. Genet..

[247] Lyst M.J., Ekiert R., Ebert D.H., Merusi C., Nowak J., Selfridge J., Guy J., Kastan N.R., Robinson N.D., de Lima Alves F. (2013). Rett syndrome mutations abolish the interaction of MeCP2 with the NCoR/SMRT co-repressor. Nat. Neurosci..

[248] Ebert D.H., Gabel H.W., Robinson N.D., Kastan N.R., Hu L.S., Cohen S., Navarro A.J., Lyst M.J., Ekiert R., Bird A.P. (2013). Activity-dependent phosphorylation of MeCP2 threonine 308 regulates interaction with NCoR. Nature.

[249] Nagarajan R.P., Hogart A.R., Gwye Y., Martin M.R., LaSalle J.M. (2006). Reduced MeCP2 expression is frequent in autism frontal cortex and correlates with aberrant MECP2 promoter methylation. Epigenetics.

[250] Kuwano Y., Kamio Y., Kawai T., Katsuura S., Inada N., Takaki A., Rokutan K. (2011). Autism-associated gene expression in peripheral leucocytes commonly observed between subjects with autism and healthy women having autistic children. PLoS One.

[251] Carney R.M., Wolpert C.M., Ravan S.A., Shahbazian M., Ashley-Koch A., Cuccaro M.L., Vance J.M., Pericak-Vance M.A. (2003). Identification of MeCP2 mutations in a series of females with autistic disorder. Pediatr. Neurol..

[252] Beyer K.S., Blasi F., Bacchelli E., Klauck S.M., Maestrini E., Poustka A. (2002). Mutation analysis of the coding sequence of the MECP2 gene in infantile autism. Hum. Genet..

[253] Lam C.W., Yeung W.L., Ko C.H., Poon P.M., Tong S.F., Chan K.Y., Lo I.F., Chan L.Y., Hui J., Wong V. (2000). Spectrum of mutations in the MECP2 gene in patients with infantile autism and Rett syndrome. J. Med. Genet..

[254] Loat C.S., Curran S., Lewis C.M., Duvall J., Geschwind D., Bolton P., Craig I.W. (2008). Methyl-CpG-binding protein 2 polymorphisms and vulnerability to autism. Genes Brain Behav..

[255] Shibayama A., Cook E.H., Feng J., Glanzmann C., Yan J., Craddock N., Jones I.R., Goldman D., Heston L.L., Sommer S.S. (2004). MECP2 structural and 3ꞌ-UTR variants in schizophrenia, autism and other psychiatric diseases: A possible association with autism. Am. J. Med. Genet B Neuropsychiatr. Genet..

[256] Coutinho A.M., Oliveira G., Katz C., Feng J., Yan J., Yang C., Marques C., Ataide A., Miguel T.S., Borges L. (2007). MECP2 coding sequence and 3ꞌUTR variation in 172 unrelated autistic patients. Am. J. Med. Genet. B Neuropsychiatr. Genet..

[257] Peters S.U., Hundley R.J., Wilson A.K., Warren Z., Vehorn A., Carvalho C.M., Lupski J.R., Ramocki M.B. (2013). The behavioral phenotype in MECP2 duplication syndrome: A comparison with idiopathic autism. Autism Res..

[258] Xu X., Xu Q., Zhang Y., Zhang X., Cheng T., Wu B., Ding Y., Lu P., Zheng J., Zhang M., Qiu Z., Yu X. (2012). A case report of Chinese brothers with inherited MECP2-containing duplication: Autism and intellectual disability, but not seizures or respiratory infections. BMC Med. Genet..

[259] Zoll B., Huppke P., Wessel A., Bartels I., Laccone F. (2004). Fetal alcohol syndrome in association with Rett syndrome. Genet. Couns..

[260] Baker S.A., Chen L., Wilkins A.D., Yu P., Lichtarge O., Zoghbi H.Y. (2013). An AT-hook domain in MeCP2 determines the clinical course of Rett syndrome and related disorders. Cell.

[261] Kim P., Park J.H., Choi C.S., Choi I., Joo S.H., Kim M.K., Kim S.Y., Kim K.C., Park S.H., Kwon K.J. (2013). Effects of ethanol exposure during early pregnancy in hyperactive, inattentive and impulsive behaviors and MeCP2 expression in rodent offspring. Neurochem. Res..

[262] Romano-Lopez A., Mendez-Diaz M., Ruiz-Contreras A.E., Carrisoza R., Prospero-Garcia O. (2012). Maternal separation and proclivity for ethanol intake: A potential role of the endocannabinoid system in rats. Neuroscience.

[263] Repunte-Canonigo V., Chen J., Lefebvre C., Kawamura T., Kreifeldt M., Basson O., Roberts A.J., Sanna P.P. (2013). MeCP2 regulates ethanol sensitivity and intake. Addict. Biol..

[264] Tunc-Ozcan E., Ullmann T.M., Shukla P.K., Redei E.E. (2013). Low-dose thyroxine attenuates autism-associated adverse effects of fetal alcohol in male offspring’s social behavior and hippocampal gene expression. Alcohol. Clin. Exp. Res..

[265] Kim P., Choi C.S., Park J.H., Joo S.H., Kim S.Y., Ko H.M., Kim K.C., Jeon S.J., Park S.H., Han S.H. (2014). Chronic exposure to ethanol of male mice before mating produces attention deficit hyperactivity disorder-like phenotype along with epigenetic dysregulation of dopamine transporter expression in mouse offspring. J. Neurosci. Res..

[266] Bekdash R.A., Zhang C., Sarkar D.K. (2013). Gestational choline supplementation normalized fetal alcohol-induced alterations in histone modifications, DNA methylation, and proopiomelanocortin (POMC) gene expression in beta-endorphin-producing POMC neurons of the hypothalamus. Alcohol. Clin. Exp. Res..

[267] Subbanna S., Nagre N.N., Shivakumar M., Umapathy N.S., Psychoyos D., Basavarajappa B.S. (2014). Ethanol induced acetylation of histone at G9a exon1 and G9a-mediated histone H3 dimethylation leads to neurodegeneration in neonatal mice. Neuroscience.

[268] Joyner A.H., Roddey J.C., Bloss C.S., Bakken T.E., Rimol L.M., Melle I., Agartz I., Djurovic S., Topol E.J., Schork N.J. (2009). A common MECP2 haplotype associates with reduced cortical surface area in humans in two independent populations. Proc. Natl. Acad. Sci. USA.

[269] Fumagalli F., Racagni G., Riva M.A. (2006). The expanding role of BDNF: A therapeutic target for Alzheimer’s disease?. Pharmacogenomics J..

[270] Couvert P., Bienvenu T., Aquaviva C., Poirier K., Moraine C., Gendrot C., Verloes A., Andres C., le Fevre A.C., Souville I. (2001). MECP2 is highly mutated in X-linked mental retardation. Hum. Mol. Genet..

[271] Watson P., Black G., Ramsden S., Barrow M., Super M., Kerr B., Clayton-Smith J. (2001). Angelman syndrome phenotype associated with mutations in MECP2, a gene encoding a methyl CpG binding protein. J. Med. Genet..

[272] Olkhov-Mitsel E., Bapat B. (2012). Strategies for discovery and validation of methylated and hydroxymethylated DNA biomarkers. Cancer Med..

[273] Montano C.M., Irizarry R.A., Kaufmann W.E., Talbot K., Gur R.E., Feinberg A.P., Taub M.A. (2013). Measuring cell-type specific differential methylation in human brain tissue. Genome Biol..

[274] Guintivano J., Aryee M.J., Kaminsky Z.A. (2013). A cell epigenotype specific model for the correction of brain cellular heterogeneity bias and its application to age, brain region and major depression. Epigenetics.

[275] Mainous A., Tanner R., Baker R., Zayas C., Harle C. (2014). Prevalence of prediabetes in England from 2003 to 2011: Population-based, cross-sectional study. BMJ Open.

[276] Volkmar M., Dedeurwaerder S., Cunha D.A., Ndlovu M.N., Defrance M., Deplus R., Calonne E., Volkmar U., Igoillo-Esteve M., Naamane N. (2012). DNA methylation profiling identifies epigenetic dysregulation in pancreatic islets from type 2 diabetic patients. EMBO J..

[277] Dayeh T., Volkov P., Salö S., Hall E., Nilsson E., Olsson A.H., Kirkpatrick C.L., Wollheim C.B., Eliasson L., Rönn T. (2014). Genome-wide DNA methylation analysis of human pancreatic islets from type 2 diabetic and non-diabetic donors identifies candidate genes that influence insulin secretion. PLoS Genet..

[278] Toperoff G., Aran D., Kark J.D., Rosenberg M., Dubnikov T., Nissan B., Wainstein J., Friedlander Y., Levy-Lahad E., Glaser B. (2012). Genome-wide survey reveals predisposing diabetes type 2-related DNA methylation variations in human peripheral blood. Hum. Mol. Genet..

[279] Bell C.G., Finer S., Lindgren C.M., Wilson G.A., Rakyan V.K., Teschendorff A.E., Akan P., Stupka E., Down T.A., Prokopenko I. (2010). Integrated genetic and epigenetic analysis identifies haplotype-specific methylation in the FTO type 2 diabetes and obesity susceptibility locus. PLoS One.

[280] Levenson V.V., Melnikov A.A. (2012). DNA methylation as clinically useful biomarkers-light at the end of the tunnel. Pharmaceuticals.

[281] Levenson V.V. (2010). DNA methylation as a universal biomarker. Expert Rev. Mol. Diagn..

[282] Papageorgiou E.A., Karagrigoriou A., Tsaliki E., Velissariou V., Carter N.P., Patsalis P.C. (2011). Fetal-specific DNA methylation ratio permits noninvasive prenatal diagnosis of trisomy 21. Nat. Med..

[283] Marsit C.J., Lambertini L., MacCani M.A., Koestler D.C., Houseman E.A., Padbury J.F., Lester B.M., Chen J. (2012). Placenta-imprinted gene expression association of infant neurobehavior. J. Pediatr..

[284] Marsit C.J., Maccani M.A., Padbury J.F., Lester B.M. (2012). Placental 11-beta hydroxysteroid dehydrogenase methylation is associated with newborn growth and a measure of neurobehavioral outcome. PLoS One.

[285] Maccani M.A., Padbury J.F., Lester B.M., Knopik V.S., Marsit C.J. (2013). Placental miRNA expression profiles are associated with measures of infant neurobehavioral outcomes. Pediatr. Res..

